# Functionalized albumin nanoparticles: A multifunctional platform for enhanced brain drug delivery

**DOI:** 10.1016/j.mtbio.2025.102616

**Published:** 2025-12-02

**Authors:** Hanan Mohammad, Maher Darwish, Gábor Katona, Ildikó Csóka

**Affiliations:** a Institute of Pharmaceutical Technology and Regulatory Affairs, Faculty of Pharmacy, University of Szeged, H-6720, Szeged, Hungary; b Department of Optics and Quantum Electronics, University of Szeged, Dóm sq. 9, 6720, Szeged, Hungary; c Department of Pharmaceutical Chemistry and Drug Control, Faculty of Pharmacy, Wadi International University, Homs, Syria

**Keywords:** Albumin nanoparticles, Brain delivery, Blood-brain barrier, Functionalization, Nanomedicine, CNS disorders

## Abstract

Albumin nanoparticles represent a unique protein-based platform for brain drug delivery, leveraging albumin intrinsic properties including exceptional biocompatibility, prolonged 19-day circulation half-life, and endogenous transport pathways via gp60 and FcRn receptors. While the blood-brain barrier restricts ∼98 % of therapeutic agents from reaching the central nervous system, functionalized albumin nanoparticles overcome this challenge by hijacking native transport mechanisms at the barrier interface. This review delivers an extensive evaluation of various methodologies for functionalizing albumin nanoparticles via covalent and non-covalent approaches aimed at improving barrier permeability, enhancing targeting precision, and enabling controlled drug release. Particular emphasis is given to ligand-based modifications that enable active receptor targeting and stimuli-responsive systems triggered by the brain microenvironment. Unlike reviews covering general nanocarrier systems, this work specifically focuses on the distinctive properties and functionalization strategies unique to albumin-based platforms. Albumin multiple reactive groups provide a versatile scaffold for functional ligand conjugation, distinguishing it from synthetic nanocarriers. Despite persisting challenges, including formulation consistency and clinical translation enhancement, functionalized albumin nanoparticles provide versatile solutions with promising advancements for treating brain tumors, neuroinflammatory disorders, and neurodegenerative diseases.

## Graphical abstract

**Figure:**
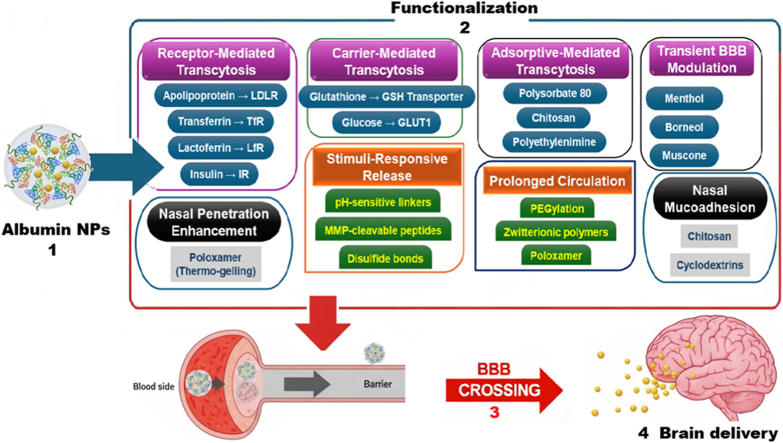

## 1 Introduction

Treatment of central nervous system (CNS) disorders faces significant obstacles due to the blood–brain barrier (BBB), a specialized protective interface that rigorously governs the exchange between the circulatory system and the brain tissue [1]. The barrier restricts ∼98 % of small molecules and virtually all large therapeutic agents from penetrating the CNS, resulting in suboptimal therapeutic effects and increased systemic toxicity [2,3]. Recently, advanced nanomaterial platforms have been developed to tackle these issues by offering tunable size, surface chemistry, and harnessing transport methods to carry treatments across the BBB. These advancements highlight the urgent need for innovative CNS delivery systems that can effectively overcome this "last frontier" in drug delivery [4].

Serum albumin is garnering substantial interest as a natural conveyance medium for drug delivery to the brain. This ubiquitous blood protein exhibits a 19-day half-life, numerous hydrophobic binding sites, and endogenous transport pathways via gp60 and FcRn receptors that enable transcytosis across endothelia [[5], [6], [7]]. Significantly, as an autologous protein, albumin is highly biocompatible and non-immunogenic. The clinical viability is exemplified by Abraxane®, an FDA-approved albumin-bound paclitaxel formulation [8]. While albumin-based carriers show promise, unmodified albumin nanoparticles (AlNPs) face key limitations in brain delivery: reticuloendothelial system (RES) recognition, weak BBB glycocalyx adsorption, premature drug release, and lack of active transport mechanisms [9,10]. Functionalization addresses these challenges by equipping AlNPs with ligands that hijack native BBB transport pathways, extend circulation, and enable stimuli-responsive drug release.

The current body of literature has extensively explored albumin nanocarriers and the targeting of the BBB; however, the intersection of these domains remains unexplored. Tincu et al. evaluated albumin-based systems for glioblastoma treatment [11], Lamichhane and Lee examined “albumin nanoscience” in cancer-targeting [12], Hornok detailed serum AlNPs production and clinical uses [13], Hoogenboezem et al. classified albumin delivery methodologies [14], and Zha et al. investigated functionalized nanomaterials traversing the BBB [15], though albumin was not specifically highlighted. In contrast to these studies, this review is the first dedicated to functionalized AlNPs as an integrated approach for brain delivery, systematically categorizing moieties by their primary mechanism of BBB transport enhancement and AlNPs modification. A structured search across PubMed, Scopus, Web of Science, and Google Scholar identified 35 original research articles meeting stringent inclusion criteria: the nanoparticle core must be albumin-based (not merely albumin-coated), and the intended application must involve brain or CNS delivery. To the best of our knowledge, this is the first review to systematically categorize functional groups according to their primary mechanisms of action in facilitating BBB penetration and enhancing therapeutic effectiveness. The detailed comparison with the aforementioned recent reviews is depicted in Table 1 to explicitly demonstrate our review novelty.

**Table 1:** Comparison of recent reviews on AlNPs for CNS/brain drug delivery.

Review	Primary Focus	Scope	BBB Mechanisms	Classification	Unique Contributions
**Current Review**	Functionalized AlNPs for CNS/brain delivery	>35 studies; All functionalization strategies; Comprehensive BBB mechanisms	RMT, CMT, AMT, EPR (systematically classified)	By BBB transport pathway + pharmacokinetic role	First systematic BBB mechanism-based classification; Quantitative efficacy comparisons; Comprehensive clinical translation roadmap
**Tincu *et al.* (2023)**	Albumin-based systems for glioblastoma	Disease-specific: Glioblastoma only	RMT only (limited coverage)	Not systematically classified	Glioblastoma-specific albumin formulations
**Lamichhane & Lee (2023)**	Albumin nanoscience for cancer targeting	Application-specific: Cancer (not CNS-focused)	Not BBB-specific	By cancer type, not mechanism	Cancer nanoscience perspective; Not brain-focused
**Hornok (2021)**	Production and clinical uses of serum AlNPs	General: Albumin NP production methodologies	General mention only; Not mechanistic	By production method, not BBB function	Production methodologies; Manufacturing focus
**Zha *et al.* (2020)**	Functionalized nanomaterials crossing BBB	Material-general: Multiple nanomaterials; Albumin not highlighted	General BBB crossing (not albumin-specific)	By nanomaterial type, not mechanism	Broad nanomaterial survey; Albumin not emphasized
**Hoogenboezem *et al.* (2016)**	Classification of albumin delivery methodologies	Methodology: Covalent vs non-covalent approaches	Not BBB-focused	By conjugation type (covalent/non-covalent)	Conjugation chemistry classification; General drug delivery

## 2 The blood-brain barrier: structure, transport, and factors influencing the passage of drug molecules

### 2.1 Structure of the BBB

The BBB is formed by brain capillary endothelial cells interconnected by tight junctions (TJs), supported by pericytes and astrocytic end-feet. Endothelial cells constitute the primary cellular component, connected by protein complexes including occludins, claudins, and junctional adhesion molecules anchored to the actin cytoskeleton [16]. Unlike peripheral endothelia, BBB endothelial cells lack fenestrations and express minimal pinocytosis, creating exceptional selectivity [15,17]. Pericytes and astrocytes are essential for BBB functions, exchanging signaling substances and communicating with brain capillary endothelial cells [18,19]. Brain-derived neurotrophic factor and hepatocyte growth factor promote TJ assembly. Conversely, cytokines and C-reactive protein disrupt it, creating a dynamic architecture that maintains CNS homeostasis but profoundly limits therapeutic access. TJs act as paracellular barriers restricting permeability to molecules <2 nm or <400–500 Da. These specialized connections form structural seals limiting paracellular transport and separating plasma membranes [20]. TJs are dynamic structures constantly formed, rearranged, and dissolved [21]. Alterations in TJs structure break the barrier and lead to neurological diseases. The causes of these alterations are multifactorial, and the underlying mechanisms under pathological conditions may potentially be manipulated to enhance drug delivery [22].

### 2.2 Transport mechanisms across the BBB

Transport across the BBB occurs through several mechanisms: passive diffusion, carrier-mediated transport (CMT), and transcytosis [23]. Transcytosis can be divided into vesicular transport and adsorption-mediated transcytosis. These transport pathways are extensively leveraged for CNS drug delivery using AlNPs [24,25]. Physicochemical features of nanoparticles determine their transport pathway and overall BBB penetration efficiency [26]. Passive diffusion allows small (<500 Da), lipophilic, non-charged molecules to cross freely through transcellular pathways. Common examples include caffeine and alcohol [27,28]. Under pathological conditions such as brain tumors, BBB disruption enables passive nanoparticle extravasation via the enhanced permeability and retention (EPR) effect [29]. Surface functionalization such as PEGylation can extend circulating half-life, enhancing chances of accumulation in leaky tumor regions. CMT employs endogenous substrate-specific transporters. Glucose transporter-1 (GLUT1) translocates glucose, mannose, and galactose across the BBB [30]. L-type amino acid transporter 1 (LAT1) mediates transport of large branched-chain and aromatic neutral amino acids [31]. The predominant expression of LAT1 at the BBB and in glioma cells provides a novel dual-targeting option for AlNPs [32].

Vesicular transport (transcytosis) involves cells engulfing extracellular material in vesicles transported to the abluminal side and released into CNS extracellular space [33]. This mechanism is essential for transporting macromolecules, therapeutic proteins, and nanoparticles, with the extent dependent on BBB physiological state [17]. Adsorptive-mediated transcytosis (AMT) occurs through electrostatic interactions between positively charged ligands and the negatively charged cell membrane, facilitated by clathrin-dependent endocytosis [38]. For AlNPs, cationization via coating with cationic polymers like chitosan or chemical modification enhances BBB penetration. However, excessive positive charge increases cytotoxicity and non-specific binding, necessitating careful balance in design [34]. Receptor-mediated transcytosis (RMT) utilizes peptide receptors on cell membranes to mediate ligand transcytosis [35]. AlNP functionalization with targeted ligands (antibodies, peptides, or aptamers) leverages RMT pathways. Albumin multiple carboxylic and amino groups provide an adaptable scaffold for such covalent attachment.

### 2.3 Factors influencing the passage of drug molecules through the BBB

Multiple factors influence BBB permeability: molecular size (<500 Da favored) [36,37], lipophilicity (optimal logP 1–3) [38,39], charge (neutral preferred; charged molecules require active transport) [40,41], and efflux pump susceptibility. Molecules with low polar surface area tend to pass through membranes. P-glycoprotein actively extrudes hydrophobic compounds [42,43], significantly reducing brain uptake efficiency [44]. Drug binding to plasma proteins reduces the amount available to cross the BBB [45], influencing free drug availability [46,47]. AlNP functionalization strategies must balance these parameters to achieve effective brain delivery.

## 3 Albumin structure and types

### 3.1 Structure of albumin

Albumin is a 66.5-kDa globular protein composed of a single 585-amino-acid polypeptide chain, organized into three homologous domains (I, II, III), each subdivided into A and B subdomains. Human serum albumin (HSA) is approximately 80 × 80 × 30 Å [48]. Seventeen intramolecular disulfide bridges confer exceptional thermal and chemical stability [49]. The protein amphipathic architecture (hydrophobic binding pockets in Sudlow sites I and II in subdomains IIA and IIIA, flanked by charged surface residues) enables simultaneous encapsulation of hydrophobic drugs and covalent conjugation of hydrophilic ligands [50,51]. Multiple reactive groups (35 lysines, 1 free cysteine, 59 glutamic/aspartic acids) serve as anchors for chemical functionalization. This balance between hydrophobic and hydrophilic regions enables albumin to bind diverse drugs with different physicochemical properties [52]. Sudlow site I (subdomain IIA) and Sudlow site II (subdomain IIIA) exhibit capacity to bind small molecules, fatty acids, and numerous therapeutic agents [53].

### 3.2 Types of albumins for drug delivery

#### 3.2.1 Human serum albumin

HSA constitutes roughly 60 % of protein in human plasma and is approved for therapeutic uses including hypoalbuminemia, sepsis, and acute respiratory distress syndrome [54]. HSA exhibits substantial aqueous solubility, biodegradability, and non-immunogenicity, rendering it ideal for drug formulations [55,56]. HSA interacts with receptors overexpressed in diseased tissues (SPARC, gp60, FcRn), providing innate targeting without additional ligands [57,58]. HSA nanoparticles overcome multidrug resistance (MDR) by using internalization pathways that bypass drug efflux pumps [59,60]. HSA also presents promise in creating theranostic agents combining diagnostic and therapeutic capabilities [61].

#### 3.2.2 Bovine serum albumin

Bovine serum albumin (BSA) shares 76 % sequence homology with HSA and exhibits a similar three-dimensional, heart-shaped structure [62]. Structurally, BSA consists of 583 amino acids (compared to 585 in HSA) and contains two tryptophan residues, while HSA has one. Although both proteins are widely used due to their affordability and accessibility, BSA displays slightly lower hydrophobicity than HSA, which can influence drug-binding affinity [63,64]. This difference has been shown to affect binding capacities, as demonstrated by Alhankawi et al., who found a positive correlation between drug hydrophobicity and its binding affinity to both albumins [65]. Moreover, subtle disparities in hydrophobicity, binding site architecture, and surface charge may further influence therapeutic outcomes and drug-binding behaviors [53,66].

#### 3.2.3 Recombinant albumin

Recombinant human serum albumin (rHSA) is synthesized using recombinant DNA technology in non-animal hosts, resulting in a product with superior purity and batch-to-batch consistency. This approach eliminates animal-sourced contaminants and blood-borne pathogen risks while minimizing immune response [66]. rHSA also addresses ethical concerns related to animal-derived proteins. Its stringent quality control and reliable production make it especially suited to pharmaceutical applications and an attractive platform for advanced nanoparticle synthesis [67,68].

## 4 Advantages and limitations of AlNPs for brain-targeted delivery

### 4.1 Intrinsic advantages of AlNPs

Even without functionalization, AlNPs demonstrate several intrinsic properties that facilitate BBB penetration and brain accumulation, clearly distinguishing them from synthetic nanocarriers. As detailed in Section 3.2.1, albumin exceptional biocompatibility and endogenous nature support immune evasion and minimize hypersensitivity and cytotoxicity compared to synthetic carriers [69,70]. The naturally prolonged plasma half-life of albumin (19 days) maintains higher circulating drug concentrations, thereby increasing cumulative brain exposure and therapeutic effectiveness [70,71]. In addition, the multiple hydrophobic pockets within albumin enable efficient encapsulation of lipophilic CNS-active drugs via non-covalent interactions, further enhancing BBB permeability [72]. Albumin also endogenously engages brain endothelial transport pathways, supporting RMT across the brain endothelium without requiring additional ligand conjugation. Under pathological conditions such as brain tumors and neuroinflammation, AlNPs accumulate more efficiently through mechanisms including SPARC binding, EPR in leaky vasculature, and preservation of nanoparticle integrity due to net negative charge at physiological pH (−15 to −20 mV) [73,74]. Furthermore, stimuli-responsive release can be engineered into AlNPs to enable site-specific drug delivery triggered by local microenvironmental factors, such as pH or enzymatic activity [[75], [76], [77]]. Notably, the nanoscale formulation of albumin confers advantages over native albumin, allowing for improved barrier penetration, controlled drug release, and enhanced delivery specificity.

### 4.2 Challenges and limitations

The application of AlNPs faces notable difficulties. A key obstacle is their limited effectiveness influenced by size, size distribution, and shape. Nanoparticles exceeding 100 nm often face challenges in extravasating into tumor microenvironments or affected areas [78]. Deep tissue penetration capacity, particularly for solid tumors, is frequently restricted due to heterogeneous biodistribution [79,80]. Organ clearance poses substantial hindrance: the spleen efficiently removes particles >200 nm, liver cells capture those 100–150 nm, while particles <5.5 nm are eliminated by kidneys [81]. This accumulation limits the capability to achieve necessary concentrations at target locations. Beyond organ elimination, albumin nanocarriers face nonspecific uptake by phagocytic cells and unwanted immune activation [63]. Furthermore, low drug encapsulation efficiency (EE%) necessitates larger drug quantities, potentially leading to rapid or compromised release [78]. To enhance stability and mitigate burst release risks, toxic cross-linking agents may be employed, introducing toxicity [82]. Additionally, insufficient regulation of drug release mechanisms and inadequate intracellular uptake continue to pose challenges [63]. Most critically, the net negative charge of AlNPs in physiological environments significantly hinders BBB traversal, as they encounter electrostatic repulsion from negatively charged proteoglycans constituting the BBB [83]. Functionalization greatly enhances albumin characteristics, addressing inherent limitations and offering significant promise as a versatile drug delivery system. Functionalized AlNPs have been effectively utilized for therapeutic applications due to alterations in chemical, physical, and biological properties, allowing improved targeting and controlled release, leading to more effective treatment modalities.

## 5 Methods for AlNPs functionalization

AlNPs exhibit a natural tendency to bind with various drugs and compounds. The introduction of a functional group into albumin can occur through two primary mechanisms: covalent conjugation, which utilizes the polar groups of albumins, and non-covalent interactions [84]. The reversible nature of non-covalent drug binding to albumin generally relies on electrostatic or hydrophobic forces [5,63,85]. The effects of these ligand modifications can influence the structures, pharmacokinetics, and therapeutic results of AlNPs [86]. Understanding how these functionalization methods impact brain delivery is crucial, as each approach offers distinct advantages for CNS targeting. Covalent modifications provide stable, long-lasting attachments suitable for sustained brain exposure, while non-covalent interactions enable reversible binding that can respond to the brain microenvironmental cues. This understanding of ligand modification may lead to advancements in various medical and therapeutic applications, including albumin-based phototherapy, drug delivery systems, and contrast agents. This section focuses on the different methods available for the functionalization of AlNPs with particular emphasis on their implications for BBB crossing and CNS drug delivery.

### 5.1 Chemical functionalization (covalent conjugation)

**Fig. 1:**
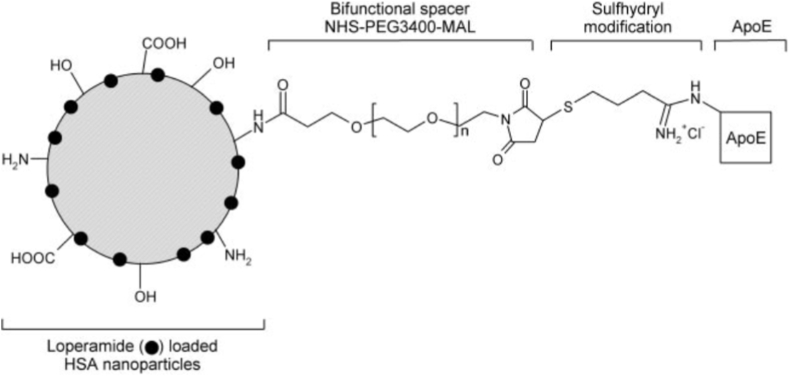
An example of covalently functionalized HSA nanoparticles.

Albumin, characterized by its high concentration of functional residues such as lysine, aspartic acid, cysteine, and serine, serves as a suitable candidate for covalent conjugation through various chemical interactions [[86], [87], [88]]. These interactions can be facilitated through amine, thiol, carboxylic acids, or hydroxyl groups, thereby allowing for the effective binding of biologically relevant compounds. Covalent coupling techniques modify albumin surface characteristics. For example, PEG incorporation enhances hydrophilicity, helping nanoparticles evade RES recognition and reduce macrophage-mediated phagocytosis. This modification also enables subsequent functionalization with other hydrophilic moieties (Fig. 1) [89].

Additionally, covalent modifications can facilitate targeted delivery through ligand binding, such as attaching folic acid to enhance affinity towards tumor cells expressing high levels of folate receptors or through interactions between immune antibodies and specific antigens within an antibody-mediated targeting framework [90]. For brain targeting applications, covalent conjugation offers several critical advantages. First, the irreversible nature of the covalent bond ensures that targeting ligands (such as transferrin, lactoferrin, or peptides) remain attached during circulation, preventing premature dissociation before reaching the BBB [23,91]. This stability is particularly important given the long transit time required for nanoparticles to accumulate at brain capillaries. Second, covalent attachment allows precise control over ligand density on the nanoparticle surface, which directly influences RMT efficiency at the BBB. Third, covalent conjugation enables the incorporation of stimuli-responsive linkers (e.g., pH-sensitive, enzyme-cleavable, or redox-responsive bonds) that can trigger controlled drug release specifically within the acidic or reductive brain tumor microenvironment, thereby enhancing therapeutic specificity and reducing systemic toxicity [92,93].

**Fig. 2:**
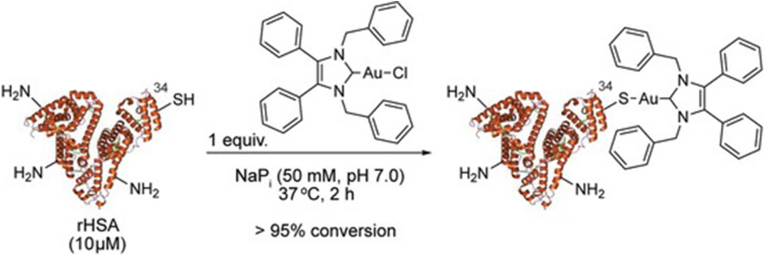
Reaction of rHSA bioconjugation with NHC∗−Au−Cl [94].

As a general example of covalent conjugation, Matos et al. established a methodology for fabricating covalent NHC∗−Au−S protein bioconjugates using rHSA and the therapeutic antibody trastuzumab. They reported a one-step, irreversible bioconjugation process that selectively targets cysteine residues. The resulting conjugate, featuring NHC∗−Au−Cl directly connected to the cysteine of rHSA, demonstrated stability in plasma (Fig. 2). As well, it was noted that the protein maintained its binding affinity to the FcRn receptor post-conjugation. This covalent conjugate is capable to enhance stability, increase the circulation half-life in the bloodstream, and improve the biodistribution of the gold-based anticancer drug [94].

### 5.2 Physical functionalization (non-covalent)

The non-covalent adsorption technique is one of the most commonly used methods for the reversible binding of proteins and various ligands or molecules. This method of interaction is clearly advantageous, as it ensures quicker access to the medication exactly when and where it is needed. Moreover, it exerts minimal impact on the structure or *in vitro* functionalities of AlNPs [95,96]. The unique secondary structure and charged functional groups present in albumin facilitate its reversible binding with various ligands and molecules through ionic bonding, coordination bonding, hydrogen bonding, Van der Waals forces, and hydrophobic interactions [97].

Utilizing ligands that non-covalently attach to albumin is a highly effective strategy for extending the elimination half-life of small biotherapeutics, thereby enhancing their pharmacokinetics through protective measures against proteolytic degradation and rapid renal filtration [63,98]. One crucial aspect of this is electrostatic interactions, a form of non-covalent binding that plays a vital role in the attachment of drugs and ligands to albumin. This occurs when substances with positive charges attract the negatively charged regions of albumin or the reverse. Though these interactions may be relatively weak, they significantly contribute to the prolonged circulation of the compounds [99].

In the context of brain delivery, non-covalent functionalization presents complementary advantages to covalent approaches. The reversible nature of non-covalent binding enables dynamic drug loading and release in response to microenvironmental changes at the BBB and within brain tissue. For instance, pH-responsive non-covalent interactions can facilitate rapid drug release in the acidic endosomal compartments of brain endothelial cells during transcytosis, or within the tumor microenvironment (pH ∼6.5–6.8) compared to normal brain tissue (pH ∼7.4) [100]. Hydrophobic interactions are particularly valuable for encapsulating lipophilic drugs that naturally exhibit higher BBB permeability, thereby combining passive diffusion with nanoparticle-mediated active transport [101]. Moreover, non-covalent adsorption of cationic polymers (such as chitosan or polyethyleneimine) can enhance AMT through electrostatic interactions with the negatively charged glycocalyx of brain endothelial cells, while maintaining the ability to release the drug once internalized. The ease of preparation and reversibility of non-covalent systems also allow for facile incorporation of multiple functional moieties simultaneously, enabling multifunctional platforms that combine targeting, imaging, and therapeutic capabilities without complex chemical synthesis [[102], [103], [104]].

**Fig. 3:**
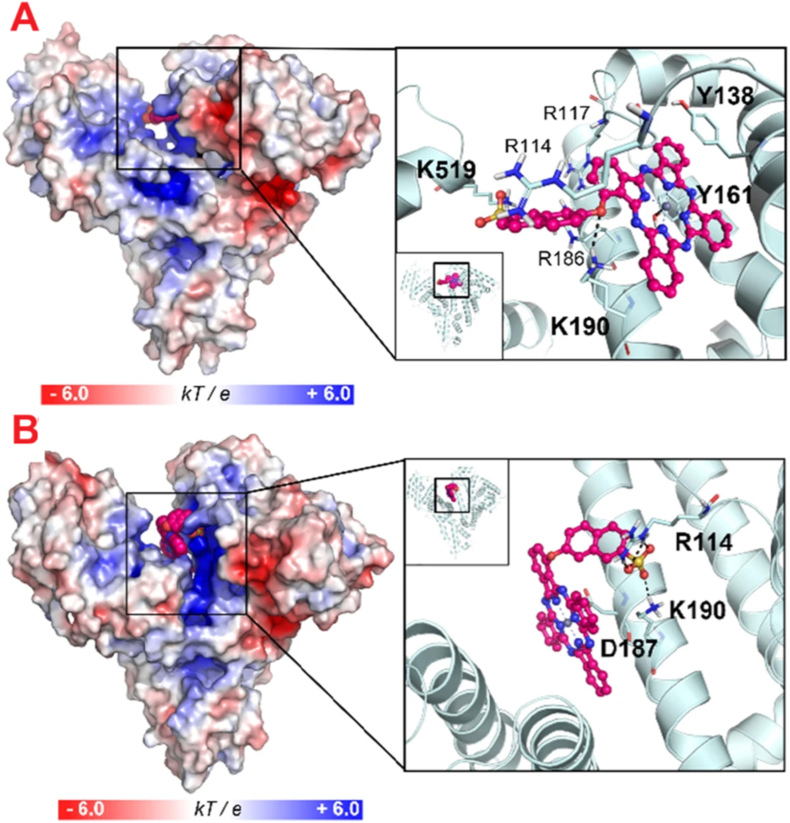
The binding mode of ZnPcS at the: A) heme site (conformer 1) and B) cleft region (conformer 2) of HSA.

The selectivity of non-covalent interactions is illustrated by Xingshu Li et al., who functionalized HSA with amphiphilic α-(6-sulfonatonaphthaleneoxyl) phthalocyaninato zinc (ZnPcS) via strong electrostatic interactions. Due to its structural similarity to the porphyrin of heme, ZnPcS was found, using blind docking simulations, to bind mainly at the heme (FA1) site of albumin, with a secondary binding mode detected within the cleft region, as illustrated in Fig. 3A and B [105].

Furthermore, hydrophobic interactions are commonly observed between albumin and various ligands and drugs. Many therapeutic agents and ligands exhibit hydrophobic characteristics, yet they can effectively dissolve in water by anchoring to albumin hydrophobic pockets. These pockets are intricately formed by a single expansive polypeptide chain that folds into a complex, three-dimensional structure, further emphasizing the effectiveness of this approach [106]. For brain delivery, the exploitation of albumin natural hydrophobic binding sites is particularly advantageous, as many CNS-active drugs are inherently lipophilic. By encapsulating such drugs within albumin hydrophobic cavities, researchers achieve dual benefits: enhanced aqueous solubility for systemic administration, and preservation of the drug lipophilic character that facilitates passive BBB diffusion once released from the carrier. This strategy effectively combines the benefits of albumin natural drug-binding capacity with nanoparticle-mediated targeted delivery.

## 6 Drug loading methods onto AlNPs

**Fig. 4:**
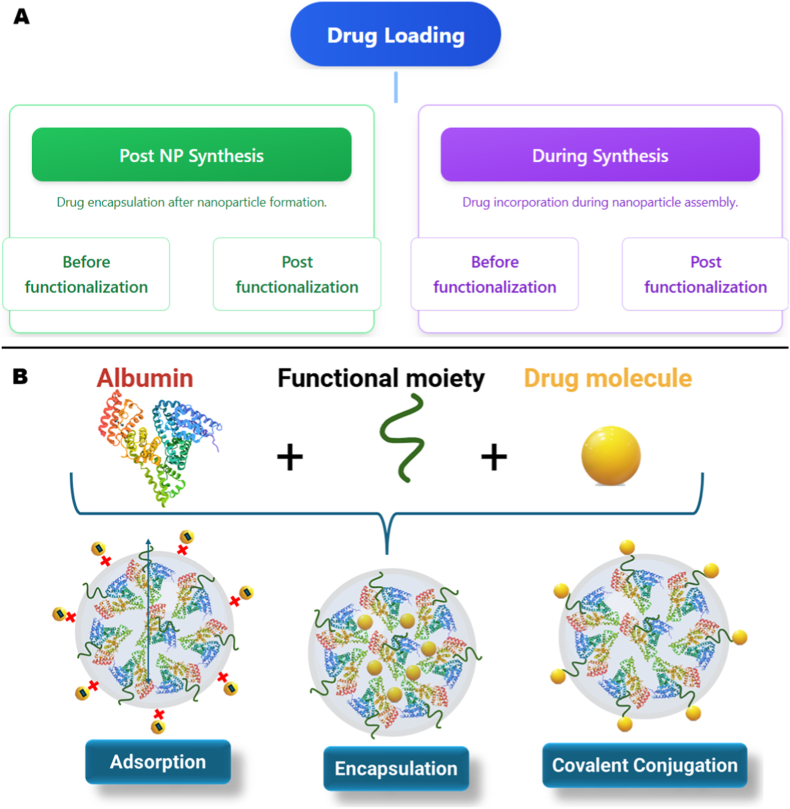
Drug loading stages and methods onto AlNPs.

The choice of drug loading method is critical for CNS delivery, as it directly influences drug release kinetics, nanoparticle surface properties, and particle size, all key determinants of BBB penetration and brain accumulation. For brain-targeted applications, the optimal loading method must balance three factors: (1) maintaining sufficient EE% to maximize therapeutic efficacy in brain tissue while minimizing systemic toxicity, (2) controlling drug release to achieve appropriate pharmacokinetic profiles for the disease target (rapid onset for acute conditions, sustained levels for chronic disorders), and (3) preserving surface properties essential for BBB transcytosis mechanisms (receptor-, carrier-, or adsorptive-mediated pathways). This section examines three primary loading approaches with specific emphasis on their implications for CNS delivery efficacy [116]. Fig. 4 shows the stages (A) and methods (B) of drug loading onto AlNPs.

### 6.1 Adsorption

A primary technique is adsorption, wherein drug molecules are affixed to the surface of the AlNP [107]. For CNS delivery, adsorption-based loading presents a trade-off. The primary advantage is simplicity and minimal drug denaturation. However, adsorption is problematic for brain targeting: weak, reversible interactions render the drug susceptible to displacement by plasma proteins during circulation, potentially resulting in premature drug loss before reaching brain capillaries. Furthermore, the BBB negatively charged endothelial glycocalyx can compete with albumin for positively charged drugs [108]. Therefore, adsorption is generally suitable only for inherently lipophilic drugs that bind strongly to albumin hydrophobic pockets, or for applications prioritizing rapid initial effect over sustained brain exposure.

Research concerning drug loading via straightforward adsorption onto AlNPs for CNS delivery remains comparatively scarce. In one study by Langiu et al., gene vectors (plasmid DNA) were adsorbed onto HSA nanoparticles, stabilized by coating with linear polyethyleneimine (PEI) to assist cellular uptake and endosomal escape in neuronal cells. These pGL3-PEI-coated HSA nanoparticles demonstrated successful transfection in cerebellar neurons *in vitro* [109]. However, no *in vivo* CNS delivery data were provided in this context.

### 6.2 Encapsulation

**Fig. 5:**
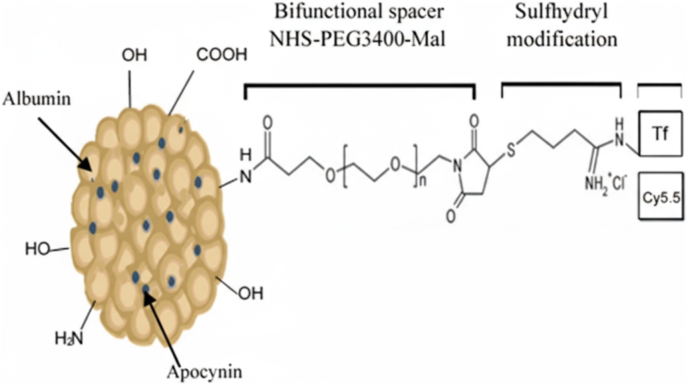
Schematic depiction of apocynin-encapsulated, transferrin receptor -targeted AlNPs utilizing the cross-linking methodology.

Encapsulation entails embedding the drug within the AlNPs structure during synthesis, shielding the drug and may regulate its release rate [107]. Common encapsulation methods include desolvation (adding ethanol or acetone to induce protein aggregation, typically with glutaraldehyde cross-linking), emulsification, nanoparticle albumin-bound (Nab) technology (clinically validated in Abraxane®), and pH-coacervation [110]. Encapsulation provides substantial advantages for CNS delivery compared to adsorption. First, the internal matrix physically shields the drug from competing plasma proteins, maintaining higher effective brain concentrations throughout circulation. Second, encapsulation stabilizes hydrophilic drugs (naturally excluded from the lipophilic BBB), achieving aqueous solubility while preserving BBB-penetrating character. Third, encapsulation enables rational control of particle size; studies demonstrate that optimal BBB penetration occurs at 50–100 nm, sufficiently large to evade renal clearance (NPs <5 nm are renally eliminated) yet small enough for endocytosis-mediated BBB crossing [111]. However, manufacturing complexity introduces batch-to-batch variability in EE% (typically 30–70 %) [112,113]. Additionally, harsh processing conditions in methods like Nab technology can compromise sensitive therapeutics [111]. A research exemplifying encapsulation for CNS delivery includes apocynin (an anti-inflammatory agent) encapsulated via desolvation and conjugated with transferrin targeting ligands (Fig. 5) [114].

### 6.3 Covalent conjugation

Covalent conjugation proves to be more advantageous here, primarily because it establishes a more stable, uniform, and reproducible attachment. Such a secure association will, on the other hand, enable a more controlled drug release through the chemically modified, stimuli-responsive AlNPs [115,116]. Consequently, it minimizes the potential adverse effects linked to drug leakage during circulation within the bloodstream [13,107]. Reports on drugs covalently bonded to AlNPs directly are rare and covalent conjugation is primarily reported for the covalent attachments of drugs to the ligands that in turn, interact with the nanoparticles as in paragraph 5.1 above.

## 7 The application of functionalized AlNPs for CNS delivery

In this review, we analyzed over thirty-five therapeutic studies utilizing functionalized AlNPs, each employing distinct functionalization strategies to address the multifaceted challenges of BBB penetration and CNS drug delivery. Although most formulations incorporate multiple functional groups (often linked via cross-linkers, though such technical details are beyond this review scope), we have deliberately focused on the fundamental functional moieties themselves and their primary mechanistic roles. These functionalization approaches primarily aim to improve pharmacokinetic profiles, prolong systemic circulation, enhance BBB transport via specific pathways (RMT, CMT, AMT, or transient modulation), and increase bioavailability at CNS target sites.

**Fig. 6:**
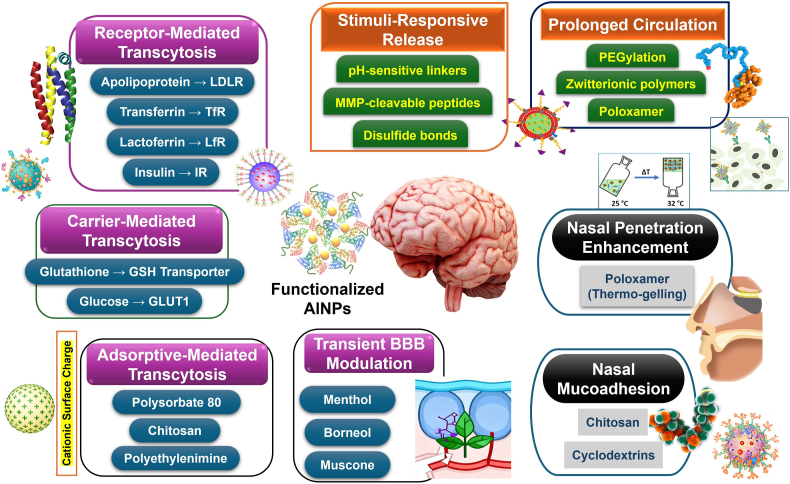
Schematic of AlNPs key functionalization strategies illustrating brain-targeting mechanisms and biobarrier overcoming approaches.

To elucidate this complex field, we have systematically categorized applications based on the primary mechanistic function that each functional group plays in enhancing nanoparticle efficacy and BBB transport (Fig. 6). This methodology inevitably involves some simplification, as real-world applications often intersect (for instance, transferrin aids in both RMT and prolonging circulation time through reduced RES uptake). When dual or multiple functions are present, we categorize formulations based on the primary therapeutic effect most consistently reported across multiple independent studies, as systematically illustrated in Fig. 6 and comprehensively detailed in the summary table provided at the end of the manuscript. This table compiles all reviewed strategies, including functionalization method, route of administration, therapeutic objective, and key outcomes.

### 7.1 Strategies to enhance BBB penetration via targeted pathways

#### 7.1.1 Receptor-mediated transcytosis

##### 7.1.1.1 Transferrin and lactoferrin receptors targeting

**Fig. 7:**
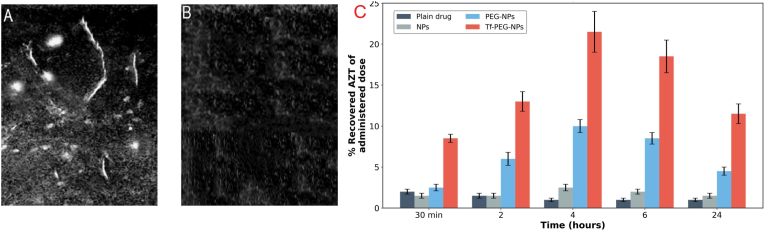
After intravenous administration for 45 min, the distribution of fluorescence within the rat brain tissue was examined utilizing a fluorescence microscope. (A) Tf-PEG-HSA NPs and (B) Plain HSA NPs; (C) *In vivo* localization of AlNPs in the brain.

The transferrin receptor (TfR), which is highly expressed on the surface of brain capillary endothelial cells to facilitate iron uptake, is one of the most extensively studied pathways for RMT of nanoparticles across the BBB [117]. Mishra et al. were among the first to translate this concept into a nanoparticle platform by encapsulating the hydrophilic antiviral azidothymidine (AZT) within long circulating, PEGylated HSA NPs and then grafting Tf-crosslinker to the NPs surface. This formulation exhibited a significantly smaller and more uniform particle size of 172.18 ± 5.2 nm with an excellent polydispersity index (PDI) of 0.232. The improved stability translated to a superior pharmacokinetic profile, with the brain AUMC for Tf-PEG-NPs (118 h μg/mL) being substantially higher than for non-targeted NPs (87 h μg/mL, 1.3-fold). Consequently, these Tf-PEG-HSA NPs achieved 21.1 ± 1.8 % of the injected AZT dose in rat brain at 4 h post IV, a dramatic enhancement over non Tf controls. Fig. 7A and B shows a clear fluorescence in the brains treated with Tf-PEG-NPs comparing to weak fluorescence detected in the control group. The drug recovery in brain of Tf-PEG-HSA was 3-folds higher compared with plain NP after 30 min (Fig. 7C) [118].

Continuing the exploration of TfR targeting, Ulbrich et al. (2009) conducted HSA NPs functionalization with either Tf itself or antibodies targeting TfR (specifically OX26 or R17217) as the functional moiety. Loperamide was loaded onto these nanoparticles, which produced robust anti-nociceptive effects in a mouse tail-flick assay following intravenous injection, achieving a maximum possible effect (MPE) of ∼92 % with lower thiolation and 100 % with higher thiolation and antibody conjugation within 15 min, sustaining the effect for up to 180 min. In contrast, control NPs decorated with non-specific IgG2a elicited only marginal responses (<20 %) [119].

**Fig. 8:**
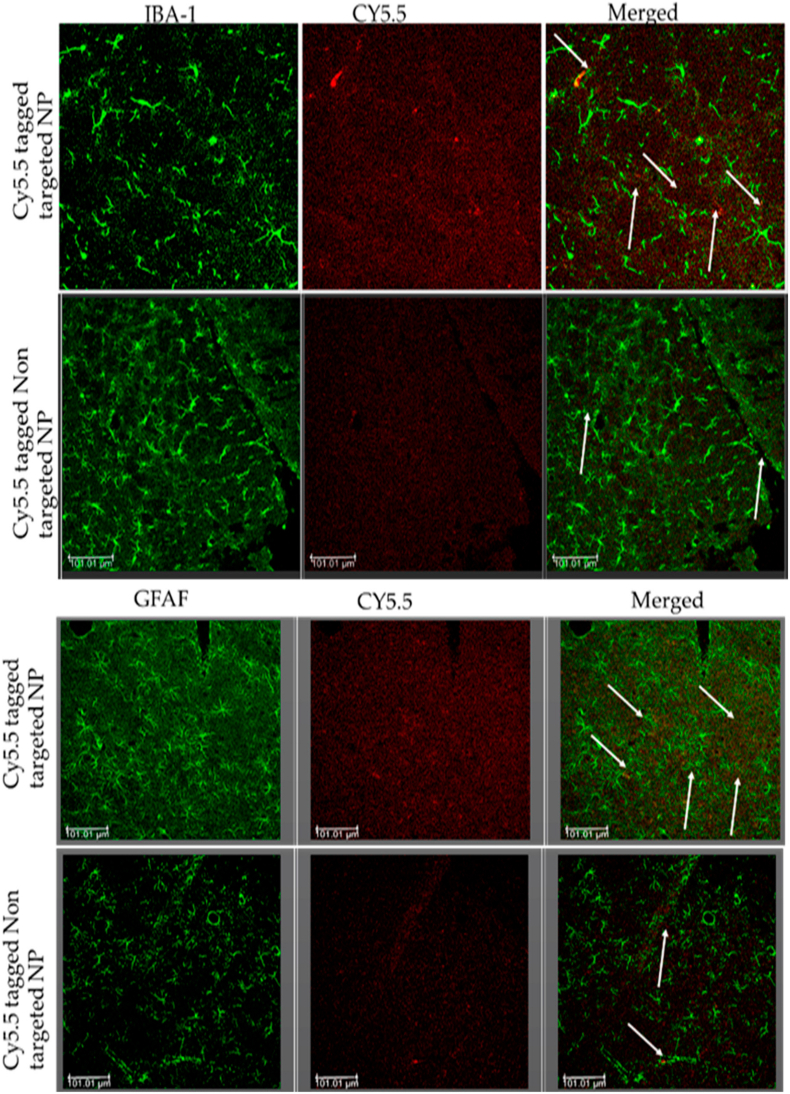
Nanoparticles penetrate the BBB and are absorbed within the brain parenchyma and glial cells, facilitated by TfR-mediated active targeting. The bTBI rats were intravenously injected with Cy5.5-conjugated apoANPs and tf-apoANPs and then sacrifice.

More recently, Perumal et al. further advanced the development of Tf-targeted systems by loading apocynin, a potent antioxidant, into their designed formulation. A 2-iminothiolane solution was used to introduce sulfhydryl groups onto Tf prior to functionalizing PEGylated BSA NPs. The formulation was designed for sustained therapeutic action, demonstrating a biphasic release pattern with an initial burst (23 % release within 30 min) followed by sustained release over 24–72 h. *In vitro*, these Tf-BSA NPs crossed human brain microvascular endothelial monolayers with up to 67 % permeability and provided superior neuroprotection; in a rodent blast traumatic brain injury (bTBI) model, they exhibited markedly higher parenchymal uptake compared to unmodified NPs (Fig. 8), which led to significant therapeutic effects, including reduced lesion volume and improved motor function recovery. The percentage of nanoparticles that crossed the BBB model increased steadily over time, showing a substantial rise in movement after 24 h of incubation compared to the initial 2-h period. When the BBB model was exposed to ethanol, migration of nanoparticles further increased, indicating that damage to the barrier enhanced their permeability. Overall, the results demonstrate that Tf-BSA NPs were capable of efficiently crossing both intact and compromised BBBs [114].

Another promising strategy for RMT drug delivery exploits the boundary of the widespread lactoferrin receptor (LfR) on both brain capillary endothelial cells and glioma cells. Pursuing this approach, Su et al. prepared BSA NPs surface-functionalized with lactoferrin and mPEG2000 as a carrier for doxorubicin (DOX). The resulting Lf-PEG-BSA-DOX NPs had a particle size of 156.3 ± 11.2 nm and a high drug loading capacity (DLC%). *In vitro* BBB and glioma co-culture models, as well as *in vivo* biodistribution studies in glioma-bearing rats, have shown that high-loading Lf-PEG-BSA nanoparticles markedly increase DOX accumulation in brain tissue at early time points and enhance cytotoxicity against both endothelial and tumor cells [120].

##### 7.1.1.2 Insulin receptor targeting

Building on the demonstrated success of Tf-targeted delivery, researchers have also explored the potential of insulin, another natural biomolecule with high RMT-based BBB crossing ability, to enhance AlNPs targeting to the brain. The strategy takes advantage of the insulin receptor high affinity and fast RMT. Stable conjugation is achieved by first thiolating insulin with 2-iminothiolane and activating AlNPs with an NHS-PEG-Mal linker. The resultant insulin-functionalized AlNPs is a stable nanocarrier optimized to penetrate the BBB efficiently [121]. In this context, Ulbrich et al. (2011) tested covalently functionalized insulin-HSA NPs loaded with loperamide. These nanoparticles produced a pronounced and sustained analgesic effect, reaching an MPE of 70 % at 60 min post-injection. Critically, control nanoparticles that were merely overcoated with insulin non-covalently failed to produce any significant effect, underscoring the necessity of stable, covalent ligand attachment for successful receptor targeting [122].

##### 7.1.1.3 Low-density lipoprotein receptor family targeting

**Fig. 9:**
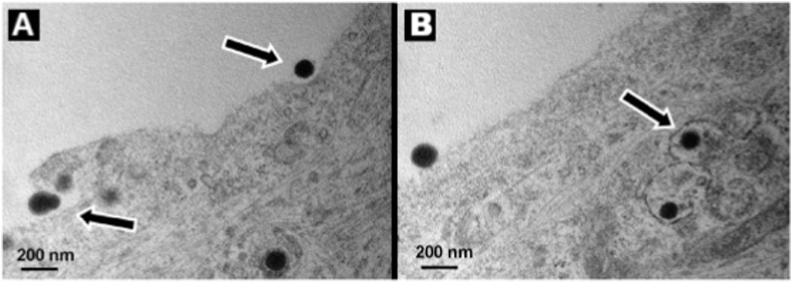
Uptake of ApoE-functionalized HSA NPs into b.End3 cells. The cell membrane undergoes invagination to encapsulate the nanoparticle. (A) and take it up by endocytosis. Inside the cells, the vesicles encapsulate the nanoparticles (B).

Apolipoproteins have been explored as ligands to engage the low-density lipoprotein receptor (LDLR) family, which is abundant on the BBB. Zensi et al. conducted two pivotal studies that offered complementary insights into the roles of specific apolipoproteins in facilitating nanoparticle brain delivery. In their 2009 study, they demonstrated that HSA NPs with covalently bound apolipoprotein E (ApoE) (249 nm, conjugated via Mal-PEG-NHS crosslinker) enabled efficient BBB penetration in SV 129 mice following intravenous administration (200 μg NPs/g body weight). Utilizing transmission electron microscopy, they precisely elucidated the transport mechanism: receptor-mediated endocytosis into cerebral endothelial cells (with visible coated pits), followed by transcytosis into brain parenchyma and uptake into neurons (Fig. 9). Importantly, control PEG-modified nanoparticles (208 nm) without ApoE failed to cross the BBB, and tight junction integrity was maintained as confirmed by lanthanum perfusion [123].

In their subsequent 2010 study, Zensi et al. explored HSA NPs with covalently bound ApoA-I, testing the formulation in both SV 129 mice (n = 8) and Wistar rats (n = 4). ApoA-I-functionalized nanoparticles successfully crossed the BBB in both species, accumulating in brain capillary endothelial cells and parenchymal tissue at 15 and 30 min post-injection. Interestingly, more nanoparticles were observed in rat brains compared to mouse brains, possibly related to differences in brain size relative to dose. While BBB crossing was confirmed by electron microscopy and tight junction integrity was maintained, the exact endocytic or transcytotic routes remained incompletely defined compared to the ApoE study. The authors proposed that ApoA-I interacts with scavenger receptor class B type I (SR-BI) on brain endothelial cells, leveraging ApoA-I role in cholesterol transport. Control PEG-nanoparticles without ApoA-I did not cross the BBB in either species [124].

Michaelis et al. later confirmed that this modification boosted the brain delivery of loperamide, raising its MPE from 20 % to 85 %, and prolonged its therapeutic action compared with polysorbate 80-coated NPs. Importantly, the new NPs preserved P-glycoprotein function and efflux integrity, and their preparation is simpler than that of polysorbate 80-coated NPs [125]. This finding was also confirmed by Kreuter et al., who obtained similar results using ApoE3, A-I, and B-100 as functional moieties. Their results showed MPE of 95 %, 65 %, and 50 %, respectively, which were statistically different from the control group (p < 0.02) [126].

Beyond direct ligand conjugation, Vishwanath et al. conducted a study in which they applied a coating of polysorbate 80 to BSA NPs preloaded with the antitumor drug paclitaxel (PTX), leveraging its capability to adsorb apolipoproteins and promote transcytosis across the BBB. This specific formulation, designated as BSA-NPs-PTX + Ps 80, demonstrated a reduced half-maximal inhibitory concentration (IC_50_) in glioma cells, an extended period of plasma exposure, and a markedly enhanced deposition of PTX in the frontal cortex, posterior brain, and cerebellum in comparison to uncoated nanoparticles or free PTX [127].

Very recently, Kurawattimath et al. developed BSA NPs loaded with palbociclib (a CDK4/6 inhibitor) and surface-functionalized with polysorbate 80 (Nps-Bsa-Palbo-Ps-80) to treat glioblastoma. The formulation exhibited optimal physicochemical properties with a particle size of 221 nm and zeta potential of 10.6 mV. In biodistribution studies in healthy male rats, Nps-Bsa-Palbo-Ps-80 demonstrated remarkable brain delivery enhancement, achieving 32-fold, 29-fold, and 9-fold increases in palbociclib concentration in the frontal cortex, posterior brain region, and cerebellum respectively, compared to free drug. The brain AUC_0-t_ was increased by 17-fold, 9-fold, and 6-fold in the same brain regions. The tissue-plasma ratio reached 1.8, 1.9, and 0.7 for frontal cortex, posterior brain, and cerebellum respectively, demonstrating substantial BBB penetration. This polysorbate 80-mediated apolipoprotein adsorption strategy successfully overcame the efflux protein limitation that normally restrict palbociclib brain penetration [128].

##### 7.1.1.4 Other receptor-based targeting strategies

**Fig. 10:**
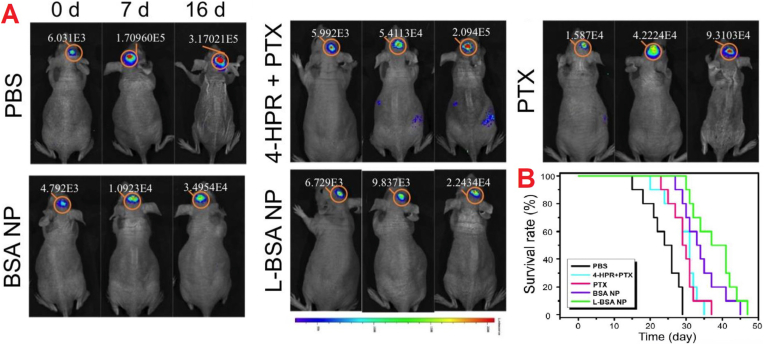
Efficacy of treatment on mice with orthotopic glioma. (A) *In vivo* bioluminescence imaging was used to track glioma growth inhibition. (B) Survival analysis curve.

Lin et al. (2016) pioneered a green synthesis method for producing functionalized BSA-NPs that were modified with the cell-penetrating peptide, low molecular weight protamine (LMWP). These nanoparticles served as carriers for the dual delivery of PTX and fenretinide (4-HPR) and were designed to target glioma by binding to the overexpressed SPARC and gp60 receptors. The enhanced structure of LMWP-modified AlNPs (L-BSA) led to significantly improved RMT-mediated penetration of the BBB and greater intratumoral infiltration depth. In orthotopic glioma models, this resulted in more effective inhibition of tumor growth. The study demonstrated that the L-BSA showed much lower bioluminescence intensity and a reduced bioluminescence area when compared to other treatment groups (Fig. 10A), indicating notable tumor reduction. Additionally, the use of L-BSA notably improved treatment outcomes, as reflected in better survival rates (Fig. 10B). The median survival times were documented as 24 days for the phosphate-buffered saline (PBS) group, 29 days for PTX alone, 31 days for the 4-HPR/PTX combination, 33 days for BSA NPs, and 37 days for L-BSA, confirming the superior effectiveness of the functionalized conjugate. The L-BSA-NPs showed substantial brain accumulation within 2 h, and the fluorescence signal peaked at 10 h (∼1.33-fold plain NP) [82].

The therapeutic application of oxytocin is notably constrained by its inherent incapacity to effectively traverse the BBB and its swift metabolic clearance, necessitating frequent dosing. To address these constraints, Zaman et al. innovatively devised a series of two distinct nanoparticle formulations for the targeted cerebral delivery of oxytocin. This study utilized BSA or poly(lactic-co-glycolic acid) (PLGA) as the core substrates, which were then functionalized with Tf or rabies virus glycoprotein (RVG). The investigators employed EDC/NHS chemistry to activate the carboxyl groups on BSA or PLGA, resulting in the creation of amine-reactive NHS esters. This facilitated the straightforward attachment of Tf or RVG to the surfaces of nanoparticles. These nanoparticles exhibited outstanding physical properties, with sizes ranging from 100 to 278 nm, and were regarded as non-cytotoxic. *In vitro* release studies demonstrated that BSA-based nanoparticles experienced an initial burst release, which is beneficial for prompt therapeutic action. RVG-conjugated BSA nanoparticles (100.1 nm) were identified as the optimal formulation due to their smallest size, favorable release profile (35 % within 6h, 50 % at 3 days), and high EE% (≥75 %). Among all formulations evaluated, RVG-conjugated BSA NPs proved most efficient in delivering oxytocin across the BBB, suggesting a promising method to enhance the therapeutic efficacy of oxytocin in the brain [129].

**Fig. 11:**
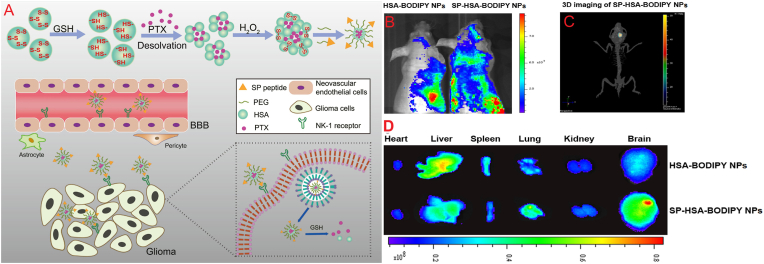
The formulation of SP-HSA-PTX NPs and their dual-targeting mechanism (BBB and glioma cells) in *in vivo* studies (A); Distribution of HSA-BODIPY NPs and SP-HSA-BODIPY NPs both *in vivo* and *ex vivo* following intravenous injection: images were captured 24 h post-injection (B), along with 3D imaging performed 24 h following the *i.v.* administration of SP-HSA-BODIPY NPs (C). Typical *ex vivo* images of the brains and organs of mice euthanized at 24 h (D).

Ruan et al. established a two-targeting drug delivery system with HSA NPs to treat glioblastoma multiforme (GBM). The intramolecular disulfide-crosslinking was used to cross-link the HSA NPs to promote redox-responsive drug release and surface-functionalize using Substance P (SP) peptide as a targeting ligand to neurokinin-1 (NK-1) receptors overexpressed on both glioma cells and brain endothelial cells (Fig. 11A). The PTX was loaded into the SP-HSA NPs, and the corresponding SP-HSA-PTX NPs demonstrated good physicochemical properties. The SP peptide served a critical role in targeting, significantly enhancing cellular uptake via receptor-mediated endocytosis and enabling BBB penetration as well as tumor localization. *In vitro*, SP-HSA-PTX NPs were more cytotoxic (IC_50_ = 121.1 ng/mL) than non-targeted NPs (IC_50_ = 294.8 ng/mL) and free PTX, and induced prominent G2/M phase arrest. *In vivo* tumor imaging in U87 glioma-bearing mice confirmed preferential tumor uptake, decreased off-target organ distribution, and better tumor inhibition (Fig. 11B–D). Most strikingly, the SP-HSA-PTX preparation increased survival in mice from a median of 21 days (free PTX) to 26 days and caused the most robust tumor tissue-induced apoptotic response [130].

Liu et al. exploited Angiopep-2-conjugated, albumin-based magnetic nanoparticles for both imaging and treating glioblastoma. Their results confirmed that the cytotoxic effects of the developed formulation on two cell lines U87MG (a human glioblastoma cell line) and 293T (a human embryonic kidney cell line) with a more pronounced effect observed in the U87MG cells. Specifically, the IC_50_ value for Angiopep-2-NPs in U87MG cells was 3-fold lower than that of non-targeted NPs, indicating enhanced cancer cell-specific killing. This suggests a degree of selectivity toward cancerous cells. However, further detailed comparisons with non-functionalized controls and comprehensive quantitative metrics were not thoroughly provided by this study [131].

Exploring further advancements in targeted delivery, folic acid (FA)-functionalized anisotropic nanoparticles have been subjected to evaluation. Lu et al. reported enhanced cellular uptake and apoptotic effects in orthotopic glioma models using FA-functionalized BSA loaded with PTX and autophagy inhibitor chloroquine (CQ). Their FA-BSA-PTX/CQ NPs showed a 10-fold higher uptake in LN229 glioma cells compared to non-targeted NPs. To quantitatively examine therapeutic efficiency, they evaluated the antitumor activity of the drug-loaded nanoparticles by FACS, and they found that cells treated with FA-BSA NPPTX/CQ displayed the highest antitumor effect, inducing an apoptosis rate of about 50 % [132].

**Fig. 12:**
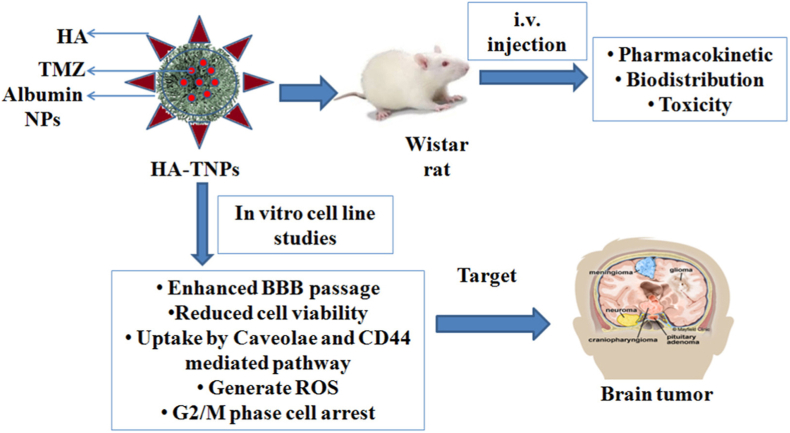
Structure, *in vivo* and *in vitro* applications of HA-TNPs.

Kudarha and Sawant linked hyaluronic acid (HA) (-COOH groups) to the surface of temozolomide (TMZ) loaded AlNPs (TNPs) using EDC-NHS carbodiimide chemistry as a conjugation agent (Fig. 12). Functionally, the HA layer served to improve the interaction with receptor in one hand and in other hand modestly reduced drug EE% from 71 % to 64 %. HA-TNPs markedly outperformed unmodified nanoparticles at crossing the BBB achieving roughly three times greater transcytosis and showed enhanced, CD44-mediated killing of U87 MG glioma cells *in vitro*. *In vivo*, they prolonged TMZ circulation (increased half-life 2-fold and AUC 1.2-fold compared with TNP), drove an 8-fold rise in brain drug levels, and cut accumulation in liver and lungs. The ratio HA-TNP to TNP in brain 1.1 and HA-TNP to TMZ 3.8 after 12 h. Crucially, no adverse hematological or biochemical effects were seen over a week of monitoring [133].

##### 7.1.1.5 Comparative analysis of the RMT strategies

Collectively, a comparative analysis of these RMT strategies highlights a spectrum of efficacy and complexity (Table 2). The Kurawattimath *et a*l. polysorbate 80-mediated apolipoprotein adsorption strategy demonstrates the highest reported brain delivery enhancement, with 32-fold, 29-fold, and 9-fold increases in frontal cortex, posterior brain, and cerebellum respectively, surpassing the HA-functionalization by Kudarha and Sawant (∼7.7-fold brain drug levels). On the other hand, while HA is naturally present in blood and tissues, a recent study revealed that polysorbate 80 may increase the deposition of pathological proteins in the brain [134,135]. Strategies focused on survival benefits, such as those using LMWP (Lin *et a*l. 2016) and Substance P (Ruan et al.), show the most compelling therapeutic outcomes with survival rates after 30 days of 100 % and 77 %, respectively. Using ApoE as a functional group resulted in higher and faster MPE compared to insulin. For example, Michaelis et al. achieved an MPE of 85 % after 20 min, whereas Ulbrich et al. (2009) reported an MPE of 70 % after 60 min. Conversely, Ulbrich et al. (2011) highlighted that insulin is significantly less expensive than apolipoproteins, making it a highly attractive alternative. However, since insulin and transferrin receptors are not brain-exclusive, comprehensive assessment of peripheral tissue distribution (e.g., liver, spleen, muscle) is crucial, but this was not addressed in their study and remains a limitation for most published work.

**Table 2:** Comparative analysis of RMT strategies in AlNPs brain delivery: ligands, quantitative results, and therapeutic outcomes.

Ligand	Study	Key Quantitative Result	Primary Advantage
Transferrin	Mishra et al.	21.1 % of injected dose in brain; 7-12-fold recovery	Optimal pharmacokinetic profile
Anti-TfR Ab	Ulbrich et al. 2009	∼92 % MPE	Highest therapeutic efficacy
Transferrin	Perumal et al.	67 % permeability; Motor recovery	Sustained neuroprotective action
Lactoferrin	Su et al.	156 nm size; High drug load	Dual BBB and glioma targeting
Insulin	Ulbrich et al. 2011	70 % MPE at 60 min	Fast RMT, alternative to TfR
ApoE (covalent)	Zensi et al. 2009	EM confirmation of neuronal uptake (249 nm NPs)	Visual proof of complete transcytotic pathway
ApoA-I (covalent)	Zensi et al. 2010	Multi-species validation (mice & rats)	Translational confidence (multi-species)
ApoE (adsorbed)	Michaelis et al.	85 % MPE (loperamide) vs 20 %	High therapeutic efficacy (non-covalent)
ApoE3, ApoA-I and ApoB-100 (covelant)	Kreuter et al.	MPE 95 % for ApoE3, 65 % for ApoA-I, and 50 % for Apo B-100	considerable antinociceptive effects after 15 min
Polysorbate 80	Vishwanath et al.	reduced half-maximal inhibitory concentration (IC_50_) in glioma	Simple, non-covalent method
Polysorbate 80	Kurawattimath et al.	32-fold increasing in frontal cortex, 29-fold in posterior brain region, and 9-fold in cerebellum	High effective brain delivery
LMWP	Lin et al. 2016	Median survival: 37 vs 31	Highest survival benefit
RVG	Zaman et al.	high EE% (≥75 %).	Burst and sustained release
Substance P	Ruan et al.	longest survival time and the greatest antitumor efficacy	Dual BBB and glioma targeting
Angiopep-2	Liu et al.	3-fold lower IC_50_ vs non-targeted	Glioma-specific targeting
FA	Lu et al.	About 50 %apoptosis rate, 10-fold higher uptake	Glioma targeting
HA	Kudarha & Sawant	1.33 8-fold rise in brain drug levels	High efficacy, prolonged circulation

Importantly, Perumal et al. were the only group to targe TfR and perform cytotoxicity testing, demonstrating that their nanoparticles were non-toxic to primary neurons an important additional layer of evidence for cellular safety. Likewise, Su et al. targeted glioma by exploiting the LfR, showing that high levels of both Lf and mPEG2000 yielded significant accumulation in the rat brain at 2 h as well as the highest cytotoxicity though broader safety and distribution profiles remain to be defined.

The Zensi et al. studies (2009, 2010) provide a unique direct comparison of two different covalently bound apolipoproteins (ApoE and ApoA-I), offering crucial mechanistic insights. The 2009 ApoE study provided the first visual confirmation of the complete transcytotic pathway to neurons, while the 2010 ApoA-I study validated the approach in multiple rodent species, enhancing translational confidence. Even though apolipoproteins achieved satisfactory MPE of over 60 % in the mentioned studies, all these investigations were limited by the lack of comprehensive *in vivo* evaluations of toxicity, tissue accumulation, and pharmacokinetic clearance. The choice of ligand is therefore a critical determinant of success, involving a trade-off between the universality of the receptor, the complexity of the conjugation chemistry, and the desired therapeutic outcome, whether it be rapid pharmacological effect, sustained delivery, or direct therapeutic impact like increased survival.

#### 7.1.2 Carrier-mediated transcytosis

Building on receptor‐targeting proteins, scientists have also used small endogenous ligands to hijack indigenous transport mechanisms at the BBB, i.e. CMT. Glutathione (GSH), a common tripeptide abundant in the brain that interacts with AMPA/NMDA receptors and employs dedicated transporters for GSH [136], has proven to be a good “shuttle” to transport AlNPs across CNS. To add this function, GSH was conjugated onto the nanoparticle surface covalently using a carbodiimide reaction via the linker 1‐ethyl‐3‐(3‐dimethylaminopropyl) carbodiimide (EDAC), enabling stable amide bond creation between GSH and surface carboxylic groups on the nanoparticle [61,137].

**Fig. 13:**
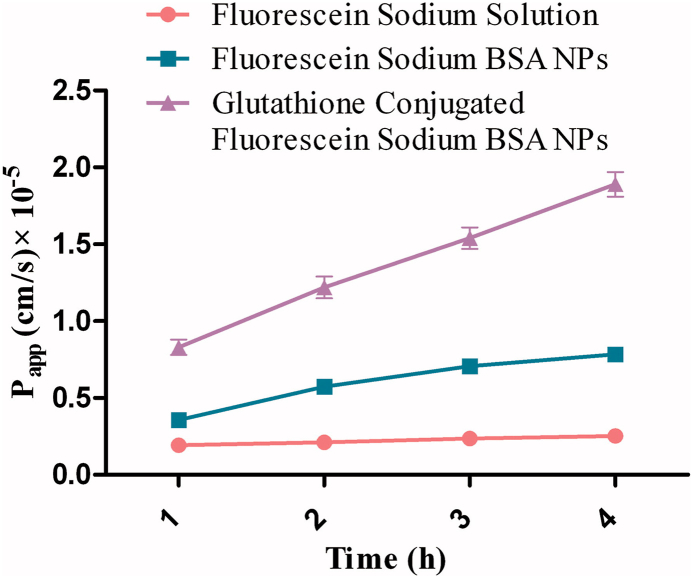
Apparent permeability of formulations loaded with fluorescein sodium across the MDCK-MDR1 cell monolayer at various time intervals.

Patel *et a*l. pioneered the use of GSH as BBB-targeting ligand by conjugating it to BSA NPs loaded with fluorescein sodium. These GSH-BSA NPs (∼270 nm with a negative zeta potential of −47mV) exhibited considerably higher permeability across MDCK-MDR1 endothelial monolayers and enhanced uptake by neuro-glial cells, resulting in a 3-fold increase in brain delivery compared to unconjugated NPs (Fig. 13). The formulation showed a biphasic release profile with an initial burst in the first 120 min and was confirmed to be non-toxic with a stable GSH coating *in vivo* [138].

**Fig. 14:**
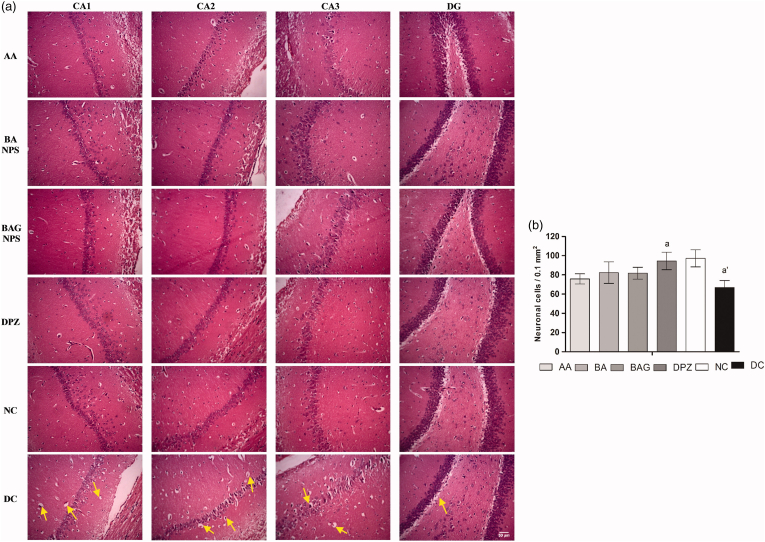
Images showcasing coronal sections of various hippocampal regions, notably CA1, CA2, CA3, and the dentate gyrus (DG), taken on the 14th day at 200 × magnification with a scale bar indicating 50 μm, are presented in (a). In (b), a graphical depiction illustrates the mean count of neuronal cells observed in the hippocampal region.

Expanding on the idea, Raval et al. showed that conjugating GSH to BSA NPs dramatically improves brain delivery of Asiatic acid. Their study in 2015 reported a 10-fold boost in brain bioavailability (drug targeting efficiency: DTE ∼627 %) at 5 h post-injection compared to free drug. Fig. 14 illustrates the histopathological examination of brain sections from rats in the disease control (DC) group, which exhibited significant intercellular spaces, inflamed cellular structures, and vacuolation when compared to the normal control (NC) group animals. The nanoparticles in this study had an average size of 228 nm and an EE% of 60 % w/w [112]. In 2018, using a Quality by Design approach, they optimized these GSH-BSA particles (∼100 nm, specifically 100.2 nm, 71.6 % EE%) and demonstrated a 7-fold increase in brain bioavailability (DTE ∼293 %) alongside marked cognitive and biochemical benefits in a dementia model. This optimization, while resulting in a lower DTE compared to their 2015 study, was geared towards achieving higher reproducibility and stability for potential clinical translation [139].

In an effort to develop advanced therapeutic strategies for neurological disorders, Duan et al. designed glucose-functionalized BSA NPs (Glu-BSA NPs) and further encapsulated a natural polyphenolic compound, procyanidin C-1 (Glu-BSA/C1 NPs). They reported that Glu-BSA/C1 NPs exhibited good stability and slow-release profile over 48 h, although there was a relatively rapid release during the first 6 h. The nanoparticles had an average size of ∼200 nm, a zeta potential of −23 mV, an EE% of 65.23 %, and a DLC% of 5.87 %. In addition, Glu-BSA/C1 NPs penetrated the BBB, accumulated in the brain by targeting GLUT1, and maintained the BBB integrity both *in vitro* and *in vivo*. Moreover, Glu-BSA/C1 NPs alleviated memory impairment of 5 × FAD mice by reducing Aβ deposition and Tau phosphorylation and promoting neurogenesis. Mechanistically, Glu- BSA/C1 NPs significantly activated the PI3K/AKT pathway and inhibited the NLRP3/Caspase-1/IL-1β pathway thereby suppressing neuroinflammation. The study highlights the advantage of targeting GLUT1, whose expression is approximately 100 times higher than the TfR in the cerebral microvasculature, leading to enhanced brain accumulation [140].

Up on comparing the studies, Raval et al. showed more nanoparticle accumulation in the brain than Patel et al., likely due to their particles being under 200 nm (while Patel et al. were over 250 nm), ideal for crossing the BBB. Additionally, Patel et al. higher negative surface charge might also have reduced brain uptake. Duan et al. targeted GLUT1, potentially causing off-target effects due to its low CNS presence, with fluorescence imaging indicating systemic distribution, raising concerns. While RMT and CMT enhance brain drug accumulation, they struggle with selectivity, competition with natural molecules, and peripheral distribution, making receptor saturation and drug load crucial for therapy and cytotoxicity. Thus, comprehensive pharmacokinetics and dosing optimization are essential in research. A detailed mechanistic and quantitative comparison of CMT approaches is presented in Table 3.

**Table 3:** Key examples of CMT for AlNPs in brain delivery.

Study	Ligand	Key Quantitative Result	Particle Size (nm)	Key Finding
**Patel *et al.***	GSH	3-fold increase in brain delivery	∼270	Established GSH as a non-toxic BBB-targeting ligand.
**Raval *et al.* 2015**	GSH	10-fold increase in bioavailability (DTE ∼627 %)	228	Highest reported brain delivery efficiency via CMT.
**Raval *et al.* 2018**	GSH	7-fold increase in bioavailability (DTE ∼293 %)	∼100	Optimized for reproducibility, highlighting efficacy vs. stability trade-off.
**Duan *et al.***	Glucose	Alleviated memory impairment in 5 × FAD mice	∼200	Successfully targeted highly expressed GLUT1 transporter.

#### 7.1.3 Adsorptive-mediated transcytosis

Nekounam et al. were the pioneering researchers to incorporate indirubin into AlNPs, as they devised an innovative hybrid chemical-mechanical methodology for the formulation of ethylene diamine CHSA nanoparticles encapsulating the hydrophobic anticancer agent indirubin, thus addressing its inadequate water solubility and restricted BBB permeability. The resulting nanoparticles achieved a high EE% of 85 % and a DLC% of 5.8 %. By chemically introducing positive charges on albumin surface (confirmed by a shift in the isoelectric point from ∼4.0 to ∼9.0) and subsequently employing high-pressure homogenization to minimize particle size, the authors observed that the initial drug release rate was slower within the polymeric structure, although the majority of the drug in both models was released within 20 h. Specifically, the release profile showed that approximately 55 % of the drug was released at 4 h, 67 % at 10 h, and 92 % at 18 h, indicating a sustained release pattern. Moreover, permeability was facilitated by the interaction of the positive charge surrounding albumin with the negatively charged surface of the brain capillary endothelium, i.e. via the AMT transport mechanism [141].

The study by Nekounam et al. is a key example of the AMT strategy, which relies on non-specific electrostatic interactions rather than specific ligand-receptor binding. This approach offers the advantage of not requiring a specific, and often expensive, targeting ligand. However, this lack of specificity is also its main drawback, as it may lead to higher uptake in other negatively charged tissues, potentially causing off-target effects. Compared to the highly efficient RMT and CMT strategies, which can achieve up to a 10-fold increase in brain delivery, the quantitative enhancement via AMT is often less pronounced, though it remains a viable and simpler alternative for enhancing BBB penetration.

#### 7.1.4 Transient modulation of BBB permeability

**Fig. 15:**
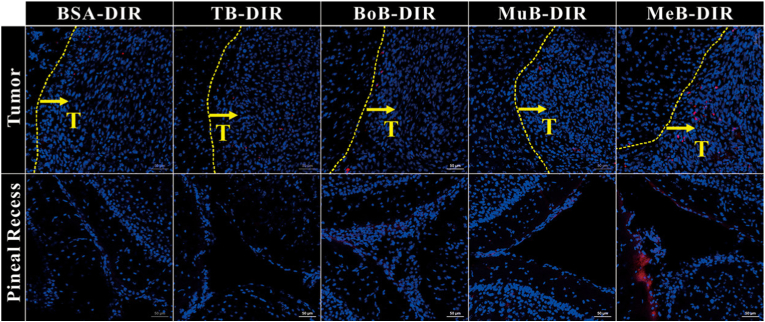
Dispersion of nanoparticles within the brains of nude mice with glioma. Red indicates DIR, blue represents cell nuclei, and the yellow line marks the glioma boundary. The yellow arrow shows the direction of glioma growth. Scale bar is 50 μm.

In addition to active targeting techniques, another promising method for improving drug delivery to the brain is the localized and precise modulation of the BBB permeability. Liang et al. indicated that natural small-molecule agents derived from traditional Chinese medicine enhance the BBB permeability. They engineered BSA NPs functionalized with four natural brain‐penetration enhancers; borneol (BoB), muscone (MuB), menthol (MeB), and thymol (TB), and labeled them with the fluorescent dye DIR to assess their distribution in healthy mouse brains. The enhancers achieved modification rates of 68.2 % (BoB), 58.5 % (MuB), and 62.3 % (MeB) on BSA nanoparticles (100–200 nm, PDI <0.3). Upon intravenous injection, MeB-DIR particles exhibited the highest cortical fluorescence intensity and were uniquely detected in both the pineal recess and cerebellar cortex, whereas BoB-DIR, MuB-DIR, and TB-DIR showed progressively diminished cortical penetration, while BSA–DIR nanoparticles without enhancers showed negligible brain accumulation (Fig. 15). The enhanced brain delivery was attributed to multiple mechanisms. Firstly, the BPE-modified nanoparticles (BoB, MuB, and MeB) induced a transient and reversible disruption of the BBB by downregulating the expression of TJ proteins ZO-1 and occludin, a mechanism not observed with the control transferrin-modified nanoparticles (TB). Secondly, MeB demonstrated the highest lipophilicity, which correlated with the highest *in vitro* BBB transport efficiency and cellular uptake into both endothelial and glioma cells. Most notably, the study unveiled a novel BBB-bypassing pathway unique to the MeB NPs, which were observed penetrating the fenestrae of capillaries in the pineal gland, a mechanism facilitated by interaction with specific MeB affinity proteins. This multi-pronged approach, paracellular (TJ modulation), transcellular (enhanced endocytosis), and direct bypass (pineal gland fenestrae), explains the superior *in vivo* glioma targeting of MeB NPs, which achieved significantly higher fluorescence intensity in the glioma region compared to the transferrin-modified nanoparticles [142].

The Liang et al. study demonstrates that natural brain-penetration enhancers, particularly MeB, can outperform conventional Tf-mediated RMT. MeB-NPs achieved the highest BBB permeability through a unique triple mechanism: (1) transient TJ protein downregulation (ZO-1, occludin), (2) enhanced lipophilicity-driven endocytosis, and (3) direct bypass via pineal gland fenestrae, a pathway not observed with Tf-modified NPs. This multi-pronged approach offers significant advantages over single-mechanism strategies. Importantly, the BBB modulation by (BoB, MuB, MeB) was transient and reversible with no tissue damage, contrasting with the neurotoxicity associated with traditional BBB disruptors like mannitol. The quantitative comparison revealed MeB > TB Tf > MuB > BoB > BSA in BBB permeability, highlighting that natural enhancers can rival or exceed ligand-based targeting while maintaining safety.

### 7.2 Strategies to optimize pharmacokinetics and drug release profile

#### 7.2.1 Achieving controlled and targeted drug release

##### 7.2.1.1 Stimuli-responsive release

**Fig. 16:**
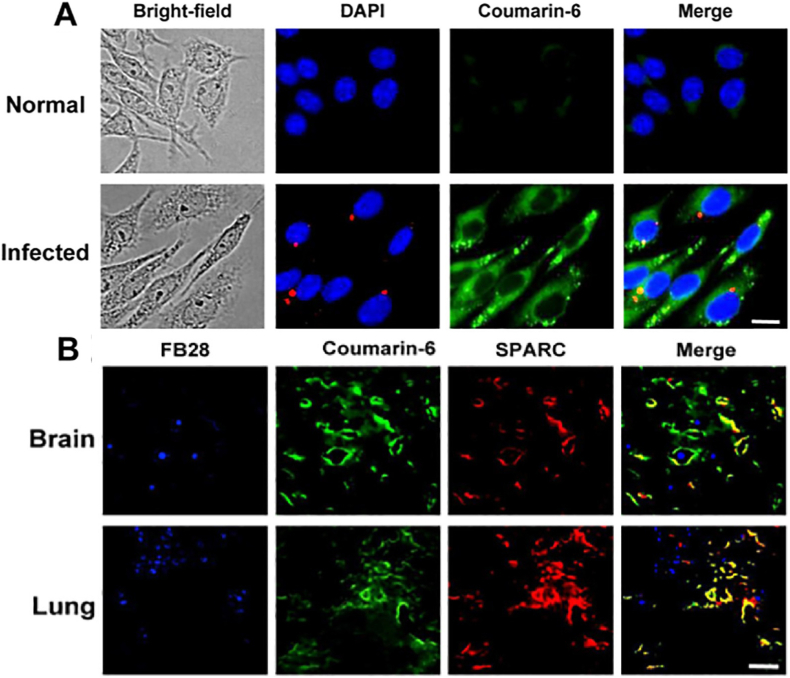
(A) BSA nanoparticle uptake in normal and infected cells by fluorescence imaging (coumarin-6, DAPI, RFP; scale = 5 μm). (B) Colocalization of coumarin-6-labeled nanoparticles and SPARC in brain and lung tissue of infected mice (scale = 20 μm).

To tackle cryptococcus neoformans infections treat challenges, Cheng et al. devised a drug delivery system using BSA NPs that encapsulated amphotericin B (AmB). The synthesized NPs were crosslinked to aggregate into larger microspheres (∼7 μm) employing a bifunctional linker, PN-PEG, targeting MMP-3. The linker consists of a BSA-binding peptide (PGNLALRPDSNS) and an MMP-3-responsive peptide (NFF-3), which demonstrated high selectivity for MMP-3 with rapid hydrolysis by MMP-3 but very slow hydrolysis by MMP-2 and MMP-9. The optimal NP/PN-PEG ratio of 4:1 achieved stable microsphere formation with exceptional EE% of 91.09 ± 1.3 % for BSA MTN/AmB, comparable to 91.13 ± 2.5 % for BSA NP/AmB, demonstrating that the assembly process does not compromise drug loading. *In vitro*, incubation with 0.15 mmol/L MMP-3 (37 °C) resulted in microsphere contraction to ∼115 nm within 2 h, as verified by scanning electron microscopy. A scrambled-peptide control showed no size reduction, verifying the specificity of the linker. After a 2-h incubation, infected cells displayed markedly stronger fluorescence signals than normal cells (Fig. 16A), and *in vivo* studies corroborated these findings, including enhanced penetration and SPARC-mediated uptake in infected lung and brain tissues (Fig. 16B). The blood concentration of BSA MTN/AmB at 24 h was twice that of conventional BSA NP/AmB, indicating significantly prolonged circulation.

The findings from the study revealed a substantial reduction in fungal colony-forming units, reduced inflammation, and notably improved survival rates, with 80 % in the treated group versus 0 % in the control group of mice. The system demonstrated more than 91 % efficiency in drug encapsulation, ensured a controlled release of the drug, showed excellent biocompatibility, and did not cause notable renal toxicity This method effectively combined passive targeting with IME-induced contraction to combat fungal infections [143]. Cheng et al. were among the first to propose the administration of nanoparticles in a macroform that can subsequently transform into the targeted nanoscale form in response to the local microenvironment. This strategy holds significant potential for reducing systemic side effects, particularly when applied to chemotherapy and radiographic imaging in cancer patients, as it enables more efficient and selective delivery of therapeutic agents or imaging contrast molecules.

##### 7.2.1.2 Modifying release kinetics

**Fig. 17:**
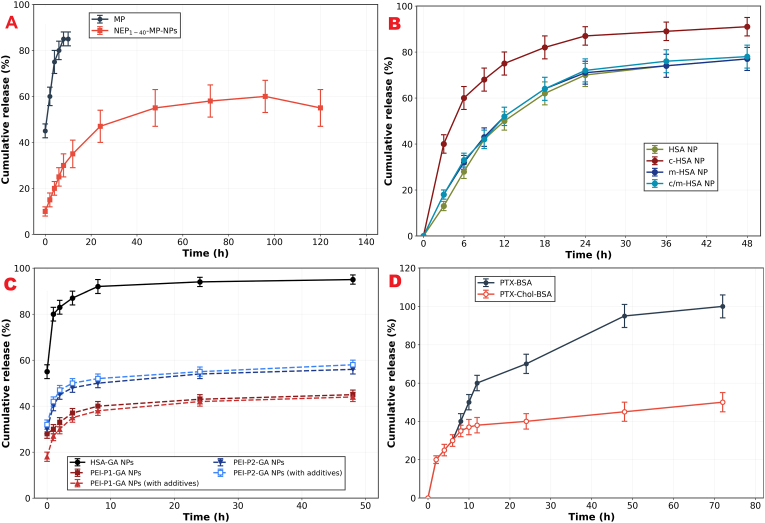
Cumulative release of: (A) MP from NEP_1-40_-MP-NPs and free MP determined; (B) DOX from HSA NPs, c-HSA NPs, m-HSA NPs, and C/m-HSA NPs; (C) GA in both unmodified and with additives, for HSA-GA, PEI-P1-GA, and PEI-P2-GA nanoparticles; (D) PTX from PTX–BSA and PTX-Chol-BSA.

Lin et al. (2019) carried out research aimed at improving the therapeutic effects of methylprednisolone (MP) for spinal cord injury (SCI) while reducing its toxic side effects. Their approach involved encapsulating MP in HSA NPs and attaching the NEP_1-40_ peptide to these nanoparticles, thus forming NEP_1-40_-MP-NPs. NEP_1-40_ is a Nogo receptor antagonist peptide that enables specific targeting of Nogo-positive cells, which are particularly relevant in SCI pathology. This approach was designed to address the poor water solubility of MP and the requirement for frequent high-dose injections. The principal findings include the successful preparation of functionalized nanoparticles with significantly enhanced cellular uptake by Nogo-positive cells relevant to SCI, demonstrating the targeting strategy validity. Additionally, NEP1-40-MP-NPs exhibited steady drug release, crucial for prolonged efficacy with minimal hemolysis (Fig. 17A). In a rat SCI model, these tailored nanoparticles were notably effective, leading to substantial enhancements in locomotor performance, reduction in bone mineral density loss, and faster recovery of the spinal cord. By day 28, the NEP_1-40_ -MP-NP group showed approximately a 1.1-fold greater improvement in locomotor function compared to the MP-NP group, with this enhancement being statistically significant (P < 0.05) [144].

Within the domain of genetic therapy, Langiu et al. developed a non-viral gene delivery system comprising HSA NPs that are cross-linked with glutaraldehyde and functionalized with a luciferase-encoding plasmid (pGL3) attached to linear PEI with a molecular weight of 22 kDa. PEI enhances DNA binding, endosomal escape, and nuclear delivery. In mouse cerebellar neurons, uptake was dose, time, and temperature-dependent, reaching ∼94 % after 72 h, with pGL3-PEI coating increasing uptake >2.5-fold compared to uncoated nanoparticles. Nanoparticles showed minimal degradation over three days. Luciferase expression followed a progressive pattern, reaching 50 % by day 2, 86.5 % by day 3, and 90.5 % by day 4, indicating efficient transfection and demonstrating the potential of AlNPs for CNS gene therapy applications [109].

**Fig. 18:**
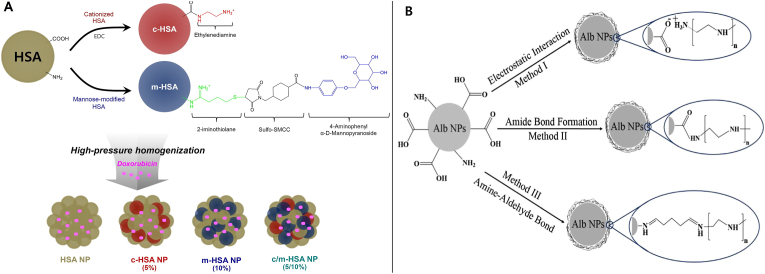
(A) Schematic representation of the C-HSA and m-HSA synthesis, alongside a depiction of the proposed strategy for forming DOX-loaded C/m-HSA NPs utilizing a high-pressure homogenizer. (B) Schematic depiction of AlNPs covered with PEI.

On the other hand, Byeon et al. engineered dual-functional HSA NPs with both cationic and mannose moieties to cross the BBB and aim glioblastoma cells. Precisely, they grafted CHSA by linking ethylenediamine to HSA native carboxyl groups, imparting a positive surface charge, and mannose-modified HSA (mHSA) by coupling mannopyranoside via a thiol maleimide reaction (Fig. 18A). The cationic modification facilitates electrostatic interaction with negatively charged BBB endothelial cells, while mannose targets glucose transporters overexpressed on both BBB and glioma cells. Their study stated that DOX-loaded C/m-HSA NPs exhibited a linear gradual DOX release over 2 days (Fig. 17B), and a prominently 4-fold lower IC_50_ than plain NP and achieved over 85 % uptake in both brain endothelial and glioma cells. In mice bearing orthotopic gliomas, these NPs homed preferentially to the tumor, suppressed growth more effectively than free or unmodified NPs, and extended median survival to 39 days (compared to 28–31 days in control groups), representing approximately a 40 % improvement in survival outcomes [145].

Mohammad-Beigi et al. tackled the challenges of low loading efficiency, incompatibility, and instability associated with the negative charges on both gallic acid (GA) and AlNPs by incorporating a cationic functional group, specifically employing PEI (Fig. 18B). They established the exploration of three separate strategies for coating AlNPs with PEI. Eventually, PEI was chosen to coat HSA NPs through covalent amide linkage enabled by N-(3-dimethylaminopropyl)-N-ethylcarbodiimide hydrochloride. This approach resulted in a significant change in zeta potential, more stable zeta potential measurements, and decreased size inconsistency. DLC% notably increased, from 13.3 % to 37.1 % when using PEI in acetone (2.8-fold increase), and from 13.3 % to 41.1 % when ethanol was used as the solvent (3.1-fold increase), demonstrating that PEI coating effectively overcomes electrostatic repulsion between negatively charged GA and albumin. As illustrated in Fig. 17C, the HSA-GA NPs exhibited an obvious burst release, releasing 86 % of GA within 1 h, compared to approximately 30 % for the other formulations. This rapid release is attributed by the researchers to the desorption of GA, which is electrostatically bound to the amine groups on the PEI shell of the PEI-HSA NPs, effectively transforming a burst release profile into a sustained release pattern through strategic surface modification [113].

To endeavor in an attempt to optimize PTX delivery, Battogtokh et al. used cholesterol hydrophobic moiety to improve the PTX loading efficacy of AlNPs and enhance colloidal stability. Cholesterol modification strengthens hydrophobic interactions between PTX and albumin, creating a more stable drug-carrier complex. Their formula (PTX-Chol-BSA NPs) showed significantly longer stay in the bloodstream than either PTX-BSA or PTX formulated in Cremophor/ethanol. As depicted in the release, PTX exhibited a release rate that was twice as slow from PTX-Chol-BSA compared to PTX-BSA over a 12-h period (Fig. 17D). Researchers noted that at the culmination of this transport mechanism, albumin-PTX complex is discharged into the subendothelial space, where albumin accumulation is likely promoted by the SPARC glycoprotein. The cellular uptake with PTX-Chol-BSA NPs was about 1.4-fold higher in B16F10 and MCF-7 cells compared with PTX-BSA NPs. A pharmacokinetic study in tumor-bearing mice showed that the area under the concentration–time curve (AUC0–8) following the administration of PTX-Chol-BSA was 1.6–2-fold higher than those following the administration of PTX-Cre/EtOH and PTX–BSA [146].

Comparative analysis of these five strategies reveals distinct approaches to modifying release kinetics for different therapeutic applications. PEI coating demonstrated the most dramatic improvement in DLC%, achieving a 3.1-fold increase for GA (from 13.3 % to 41.1 %), while simultaneously transforming burst release (86 % in 1 h) into sustained release (∼30 % in 1 h) through electrostatic binding. Cholesterol modification offered a complementary approach, slowing PTX release by 2-fold over 12 h through enhanced hydrophobic interactions. The dual-modification strategy employed by Byeon et al. combined cationic charge for BBB penetration with mannose for tumor targeting, achieving >85 % cellular uptake in both brain endothelial and glioma cells and extending median survival from 28-31 days–39 days (40 % improvement). Peptide-based targeting (NEP_1-40_) provided receptor-specific delivery for spinal cord injury, while PEI-mediated gene delivery achieved remarkable transfection efficiency (90.5 % by day 4) with 94 % cellular uptake after 72 h. These findings demonstrate that release kinetics can be precisely tuned from rapid burst to sustained release through strategic surface modifications, with the choice of strategy depending on the therapeutic goal: sustained release for chronic conditions (SCI, cancer), controlled release for avoiding toxicity (GA), or rapid cellular uptake for gene therapy. The range of stimuli-responsive release systems and their experimental performance is shown in Table 4.

**Table 4:** Comparative analysis of different strategies for modifying release kinetics in AlNPs.

Study	Modification Strategy	Release Pattern	DLC%	Key Outcome
**Lin *et al.* 2019**	NEP_1-40_ peptide	sustained release without a significant early burst	7.32 % ± 0.71	Faster SCI recovery
**Langiu *et al.***	PEI (22 kDa)	N/A (gene delivery)	N/A	90.5 % transfection (Day 4)
**Byeon *et al.***	Cationic + Mannose	Linear gradual (2 days)	HSA 5.9 ± 1.5, c-HSA, 4.9 ± 1.4, m-HSA 5.7 ± 1.7, and c/m-HSA 6.3 ± 0.9 %	39 days survival (vs. 28–31)
**Mohammad-Beigi *et al.***	PEI coating	Sustained (∼30 % in 1h)	41.1 % (vs. 13.3 %)	3.1 × loading increase
**Battogtokh *et al.***	Cholesterol	2 × slower (12h)	40 %	Prolonged circulation

#### 7.2.2 Prolonging systemic circulation (half-life extension)

In the early 1970s PEGylation emerged as versatile benefits nanoparticle modification. This process, involves covalently linking a peptide chain to high molecular weight chains of PEG [147]. It serves two main purposes: concealing antigenic determinants, thus evading antibody detection, and increasing protein size to hinder kidney [148]. PEG is a non-toxic, non-immunogenic, hydrophilic polymer that resists biodegradation. Its flexibility allows for easy attachment to therapeutic agents, and PEGylation improves protein absorption by increasing solubility in water and protecting against proteolytic degradation [149].

**Fig. 19:**
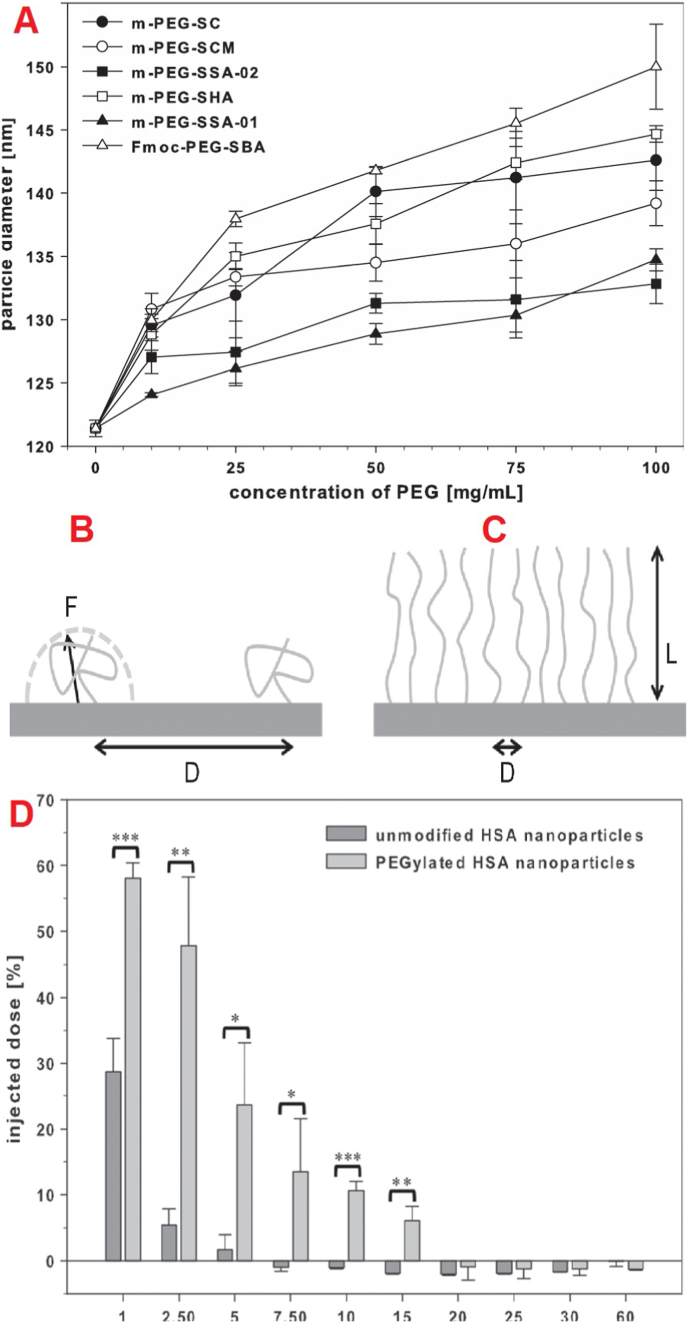
(A) PCS analysis of HSA nanoparticles with and without PEGylation. (B) Depiction of the 'mushroom' configuration and (C) the 'brush' configuration of PEG chains present on a nanoparticle surface. (D) Pharmacokinetic profile (blood concentration of nanoparticles) of non-PEGylated and PEGylated HSA nanoparticles in mice, represented as the percentage of injected dose over time post-injection.

Fahrländer et al. first showed that covalent attachment of various PEG-NHS esters to HSA NPs produced a clear, concentration dependent increase in mean particle diameter (Fig. 19A), while zeta potentials remained around −40 mV, indicating that PEGylation and loss of surface amino charge counterbalanced each other. Model calculations of grafting density versus Flory radius indicated a 'brush' conformation of surface PEG chains, favorable for steric stabilization, which is critical for preventing protein adsorption and prolonging circulation time. (Fig. 19B and C). Finally, *in vivo* pharmacokinetics in mice showed that PEGylation extended plasma half-life from 0.56 min (unmodified) to 2.20 min (PEGylated) a 4-fold increase in circulation time (Fig. 19D). This substantial improvement demonstrates that even modest increases in circulation time can significantly enhance the therapeutic window and biodistribution profile of AlNPs. A general note is that researchers incorporate usually an additional functional moiety alongside PEG to modify AlNPs, aiming to combine the advantages of PEG with supplementary benefits [150].

Wang et al. embarked on research to achieve the goal of designing nanoparticles that can deliver a greater quantity of drugs to tumors and maintain a prolonged presence at the site, thereby overcoming the challenge of rapid elimination by the RES system and insufficient accumulation at target sites. Their research concentrated on functionalized BSA NPs, particularly BSA-poly(N-3-acrylamidophenylboronic acid) (BSA-PAPBA) NPs, which were surface-modified with various grafted copolymers, including PEG, poly(carboxybetaine) (PCB), and poly(2-methacryloyloxyethyl phosphorylcholine) (PMPC). These grafted copolymers had similar degrees of polymerization (DP ∼45) and grafting numbers, ensuring a fair comparison. The coating layer thickness was homogeneous across all formulations with zeta potential shifting from −55 mV to ∼+25 mV after coating. The most significant outcomes of this functionalization were a considerable extension of the nanoparticle circulation time within the bloodstream and a notable improvement in tumor uptake, with drug accumulation at the tumor site surpassing 10 % of the injected dose per gram of tumor. Quantitative pharmacokinetic analysis revealed that the half-life of DOX in blood circulation was 12.4 h for BSA-PAPBA NP. They attributed this increase in half-life due to positive charged nanoparticles could prevent the protein adsorption *in vitro* and *in vivo*, and have a half-life of more than 10 h in blood. Tumor accumulation studies demonstrated that DOX concentration reached 8.3 ± 3.6 % ID/g for BSA-PAPBA NPs at 8 h post-injection. Crucially, the PMPC-functionalized nanoparticles exhibited superior performance regarding tumor accumulation and anti-cancer efficacy, thereby establishing these surface-modified nanoparticles as promising candidates for highly effective targeted drug delivery systems. *In vivo* antitumor studies revealed that tumor volume increased only 4-fold for PEI-PMPC NP and 5-fold for PEI-PEG NP on day 15 compared to day 1, dramatically lower than the 25-fold increase for BSA-PAPBA NP and 105-fold for free DOX. Survival analysis showed that 90 % of mice treated with PEI-PMPC NP survived to day 37, compared to 80 % for PEI-PEG NP and 60 % for PEI-PCB NP, with median survival exceeding 40 days versus only 19 days for free DOX [151].

**Fig. 20:**
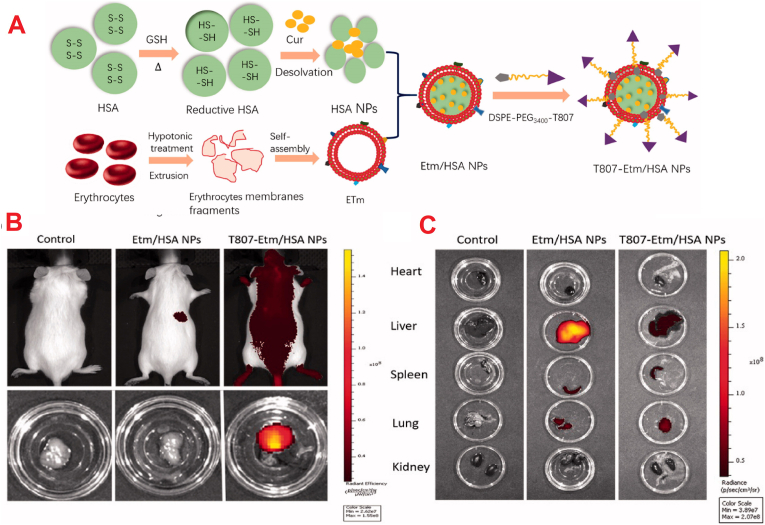
(A) The *in vitro* preparation of T807-ETm/HSA-Cur NPs. Evaluation of *in vivo* brain-targeting capability. Distribution analysis of DIR-labeled NPs in mouse brains (B) and various organs (C) via an IVIS® Spectrum.

Apart from PEG and to significantly extend the systemic half-life and enhance brain accumulation of therapeutics, Gao et al. engineered erythrocyte‐membrane-coated HSA NPs and inserted DSPE-PEG3400-T807 (Fig. 20A), which increased the EE% and exhibited sustained curcumin release over 72 h. This biomimetic approach leverages the natural stealth properties of erythrocyte membranes to evade immune recognition while incorporating T807, a tau protein-targeting ligand, for brain-specific delivery. *In vivo* studies revealed robust brain accumulation within 30 min, with minimal liver or spleen uptake (Fig. 20B and C) [152]. The erythrocyte membrane coating conferred a longer circulation half-life and reduced immunogenicity compared to synthetic material-based nanocarriers. Notably, T807-ETm/HSA NPs accumulated rapidly and specifically in the brain within 30 min, while ETm/HSA NPs without T807 showed no significant brain uptake. The T807-modified nanoparticles also demonstrated reduced distribution to the liver and spleen, confirming the dual advantage of biomimetic stealth and active targeting.

Gao et al. demonstrated exceptional brain accumulation, accompanied by an extended circulation half-life and reduced immunogenicity compared to nanocarriers composed of synthetic materials. However, their method required orthotopic administration, and the isolation of erythrocyte membranes introduce biological variability that could compromise reproducibility and scalability. In contrast, Wang et al. conducted a comprehensive comparative study of surface functionalization strategies (PEG, PCB, and PMPC), but their research lacked detailed mechanistic insight into how these coatings interact with tumor and endothelial cell receptors. Meanwhile, Fahrländer et al. focused primarily on optimizing the PEGylation process rather than assessing its therapeutic potential. Although their findings demonstrated a fourfold increase in nanoparticle circulation half-life, they did not include drug loading, release kinetics, or pharmacokinetic analysis, which could have significantly influenced the interpretation of the results.

### 7.3 Strategies for alternative administration routes: nose-to-brain delivery

The intranasal route for administration presents considerable benefits for patients and healthcare providers. This route permits circumvention of the BBB, facilitating direct drug delivery to the CNS [153]. The technique ensures prompt pharmacological action owing to the extensive vascularization within the nasal cavity, resulting in swift absorption into the bloodstream [154]. Being non-invasive and straightforward to execute, intranasal administration is particularly suitable for patients who experience difficulties with oral or injectable therapies. It also mitigates potential systemic side effects and curtails first-pass hepatic metabolism, thereby enhancing dosage efficiency and therapeutic outcomes [155,156]. This method demonstrates significant promise in targeting the CNS, as exemplified by compounds such as dopamine, levodopa, and certain proteins [[157], [158], [159]]. The primary rationale for incorporating functional groups into albumin for nose-to-brain delivery is to augment mucoadhesion and nasal mucosal penetration. The subsequent paragraphs will elaborate on both mechanisms.

#### 7.3.1 Enhancing mucoadhesion

**Fig. 21:**
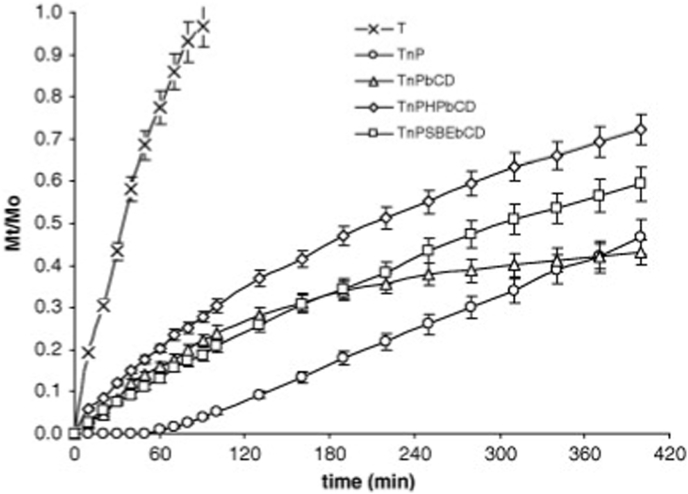
Permeation patterns (average ± SD, n = 3) of tacrine hydrochloride derived from contained nanoparticles.

Luppi et al. inspected three cyclodextrin types, namely, beta cyclodextrin (bCD) sulfobutylether-beta-cyclodextrin (SBEbCD), hydroxypropyl-beta-cyclodextrin (HPbCD), as modifying agents to produce physical interactions with albumin before evaporating the desolvation solvent. Their results showed that SBEbCD significantly improved drug loading compared to other cyclodextrin variants and non-functional BSA NPs. Furthermore, HPbCD exhibited comprehensive permeation within a time frame of 100 min, as evidenced by Fig. 21. The drug release demonstrated a prolonged duration, with SBEbCD indicating a diminished release rate due to its enhanced affinity for tacrine. This study utilized a cold setting desolvation method, which avoided the use of harmful glutaraldehyde and organic solvents. Moreover, they noted that nanoparticles containing HPbCD increased their mean size 10-fold after 24 h, evidencing enhanced mucoadhesive properties through physical interactions with adhesion strength measured at 20 dyne for nP, 29 dyne for nPbCD, 65 dyne for nPHbHPbCD, and 30 dyne for nPbSBEbCD, demonstrating that HPbCD achieved a 3.25-fold increase in mucoadhesion compared to pristine nanoparticles [160].

Alternatively, Piazzini et al. executed a comprehensive study to assess the significant effects of chitosan coating on the efficacy of HSA NPs, specifically targeting improvements in nasal drug delivery systems. The positive charge inherent to chitosan promotes electrostatic attraction with negatively charged epithelial cells of the nasal lining. The interactions play an important role in increasing the retention of the drug in the nasal cavity for prolonged duration, promoting the absorption of moisture from the mucus in nanoparticles, thereby increasing its mucoadhesive nature. Sulforhodamine B sodium salt (SulfB), which is hydrophilic in nature, was used in this study as a model drug. Turbidimetric analysis was conducted to assess the mucoadhesive behavior of the nanoparticles. The findings revealed that the absorbance difference (ΔA) of CS-HSA NPs was seven times greater after 30 min and ten times greater after 60 min compared with uncoated HSA NPs. This substantial increase in mucoadhesion was attributed to strong electrostatic interactions between the positively charged amino groups of chitosan and the negatively charged carboxyl and sulfate groups of mucin. Consequently, this interaction led to a 2-fold increase in the apparent permeability coefficient (Papp) after 4 h compared with non-chitosan-coated nanoparticles. Beyond enhancing mucoadhesion, the developed nanoparticles also demonstrated the ability to transiently open TJs between hCMEC/D3 cells by reducing ZO-1 expression levels, thereby facilitating molecular transport across the barrier [161].

#### 7.3.2 Enhancing nasal penetration

**Fig. 22:**
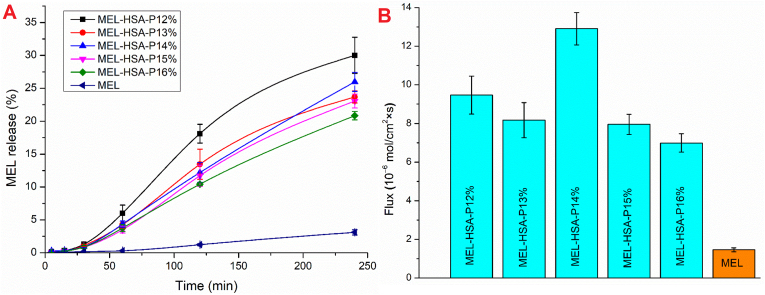
(A) The dissolution behavior of MEL-HSA-P407 formulations *in vitro* is compared to that of the initial MEL. (B) Comparison of PAMPA-BBB permeability fluxes between MEL-HSA-P407 formulations and initial MEL.

Katona et al. developed a nanoparticulate formulation of meloxicam-HSA (MEL-HSA) exploiting poloxamer 407 (P407) to boost the formulation resistance to mucociliary clearance through a sol-gel transition on the nasal mucosa, thereby facilitating better drug absorption. The authors noted that the developed formulation showed higher adhesive work than NaHA, which they attributed to a slight increase in the number of functional groups forming mucoadhesive bonds at the interface. Characterization of the nanoparticles indicated that formulations with 12–15 % w/w P407 satisfied the criteria for effective intranasal administration (Fig. 22A). The optimized formulation (MEL-HSA-P14 % containing 14 % w/w P407) exhibited a Z-average in the range of 180–304 nm, narrow PDI (0.193–0.328), zeta potential between −9.4 and −7.0 mV, and hypotonic osmolality (200–278 mOsmol/L), all of which predict enhanced drug absorption through the nasal mucosa. They reported that the small Z-average, narrow PDI, appropriate zeta potential, and hypotonic osmolality (200–278 mOsmol/L) of MEL-HSA nanoparticles indicate an enhancement in nasal drug absorption. The developed formulation significantly improves the permeability of MEL across the BBB 4-fold comparing with free MEL, as demonstrated by PAMPA-BBB assays (Fig. 22B) and confirmed through permeation studies using RPMI 2650 human endothelial cells, and the cellular studies provided that there was no damage following a 1-h exposure to final formula in RPMI 2650 human endothelial cells, with junctional proteins ZO-1 and β-catenin remaining intact, confirming the safety of the formulation for nasal administration [162].

In summary, AlNPs hold strong potential for brain-targeted drug delivery via the olfactory pathway, allowing efficient penetration into brain tissue. However, successful design requires careful optimization of particle size (ideally 20–200 nm) and zeta potential to balance mucoadhesion and uptake without impeding diffusion or promoting rapid clearance [[163], [164], [165]]. Comparative results show that the Katona et al. formulation crosses both the nasal mucosa and the BBB; Luppi et al. crosses only the nasal mucosa; Piazzini et al., while strongly mucoadhesive, remains largely retained in the nasal mucus due to high positive surface charge. Lack of *in vivo* imaging means definitive conclusions about BBB penetration for some systems remain elusive. Thus, future research with fluorescence or radiolabel imaging is essential to validate BBB permeability and clinical translatability. Key nasal delivery strategies, including mucoadhesion and penetration enhancement, are summarized in Table 5.

**Table 5:** Comparative analysis of enhancement strategies for nose-to-brain delivery.

Study	Modification	Mucoadhesion	Size	Zeta	Permeability (*ex vivo*)
**Luppi *et al.***	Cyclodextrins (HPbCD)	65 dyne (3.25 × )	<300	∼ −10 ± 0.8	N/A
**Piazzini *et al.***	Chitosan coating	7-fold (30 min), 10-fold (60 min)	261 ± 8	+45 ± 1	2-fold
**Katona *et al.***	Poloxamer 407 (14 %)	Higher adhesive work	176 ± 3	−7.9 ± 0.3	4-fold

## 8 Limitations, challenges, and future perspectives

This review underscores that AlNPs represent a promising and versatile platform for overcoming the challenges of drug delivery to the brain. By leveraging the inherent advantages of albumin. In combination with sophisticated functionalization strategies, AlNPs have demonstrated significant potential for crossing the BBB and achieving targeted drug delivery as collectively shown in Table 6. The accumulating preclinical evidence supports their therapeutic utility in a range of CNS disorders, including glioblastoma, neuroinflammation, and neurodegeneration. However, the translation of these promising findings into clinical practice is contingent upon addressing a number of significant limitations and challenges.

**Table 6:** Detailed summary of all functionalization strategies, their intended aims, experimental outcomes, and therapeutic implications.

Used AlNPs	Drug (Loaded Material)	Functional moiety(s)	Functionalization step	Drug loading stage	Route of administration	Main result(s)	REF.
HSA	AZT	PEG, Transferrin, Glutaraldehyde cross-links	PEGylation pre-NP; Transferrin anchoring post-NP	Incorporated in internal phase of w/o albumin emulsion	IV	Transport by amino acid portals present on BBB.	[118]
HSA	Loperamide	NHS-PEG-MAL-5000 linker; Transferrin; OX26/R17217 antibodies	After NP preparation	After NP preparation & functionalization	Tail vein injection	TfR-mAb-coupled HSA NPs achieved strong anti-nociceptive effects, whereas IgG2a-modified HSA NPs were not able to transport this drug across the BBB	[119]
BSA	Apocynin	Tf	After NP preparation	Apocynin in BSA solution pre-desolvation	IV	brain parenchyma and glial cells, particularly accumulated near microglia and astrocytes. By TfR reduce neuronal death	[114]
BSA	DOX	mPEG2000, Lf	After NP preparation	DOX in BSA solution pre-desolvation	IV	brain targeting by LfR P_e_ in BBB 9-fold and t_1/2_ 1.6-fold higher than plain	[120]
HSA	Loperamide	Insulin; Anti-insulin receptor antibody (29B4); NHS-PEG-MAL-5000	After NP preparation	After NP preparation & functionalization	IV	targeting insulin receptor insulin is much less expensive than the Apo Insulin alone (non-covelantly) was not able to achieve any loperamide brain delivery.	[122]
HSA	–	APO E	After NP preparation	_	IV	transported into the brain endothelial cells parenchyma by LDL	[123]
HSA	–	Apo A-I	After NP preparation	_	IV	Apo A-I seems to prefer the scavenger receptors in the brain capillary endothelial cells	[124]
HSA	Loperamide	ApoE	After NP preparation	After NP preparation (incubation)	IV	Only APO E 3 mediated brain transport vs APO E1 and APO E2 by LDL-R, LRP receprors	[125]
HSA	Loperamide	Apo E3, A-I, B-100	After NP preparation	After NP preparation & functionalization	IV	APO E and Apo B by LDL and LRP Apo A-I by scavenger receptor SR-BI at BBB	[126]
BSA	PTX	Polysorbate 80	_	_	IV	Lower IC_50_ in glioma cells; Increased PTX stability	[127]
BSA	palbociclib	Polysorbate 80	After NP preparation	During desolvation	IV	enhancing palbociclib distribution over plain NP in frontal cortex and posterior brain regions.	[128]
BSA	PTX, Fenretinide	LMWP peptide	Before NP preparation	Drug in ethanol pre-desolvation	IV	albumin-binding proteins (gp60 and SPARC) receptor	[82]
BSA	Oxytocin	Tf; RVG	After NP preparation	Oxytocin in BSA solution pre-desolvation	_	Tf and nicotinic cholinergic receptors on the BBB initial burst followed by a sustained release	[129]
HSA	PTX	Substance P peptide; Disulfide bonds	SP pre-NP; disulfide post-NP	PTX in ethanol (desolvant)	IV	Improved stability tumor targeting; GBM	[130]
BSA	Carmustine	Angiopep-2, SPIO, ICG	Post-NP via EDC	SPIO/ICG in BSA pre-desolvation; BCNU in alcohol	IV	LRP receptor targeting (BBB/GBM); preferential accumulation in tumor sites by NIRF/MRI in a preclinical model and superior therapeutic activity against GBM.	[131]
BSA	PTX-CQ	FA	After NP preparation	Post-functionalization	_	inducing glioma cell apoptosis enhancing the sensitivity of glioma stem cells to PTX treatment.	[132]
HSA	TMZ	HA	After NP preparation	TMZ in HSA solution pre-desolvation	Tail vein injection	Brain target via endocytosis and CD44 receptor mediated uptake	[133]
BSA	Fluorescein sodium	GSH (EDAC coupling)	After NP preparation	Fluorescein sodium suspended in BSA solution pre-desolvation	IV	NMDA and AMPA receptors Accumulate in brain 3-fold and apparent permeability (Papp) 2.4-fold higher than plain NP	[138]
BSA	Asiatic acid	GSH (EDAC coupling)	After NP preparation	Asiatic acid in ethanol (desolvant)	IV	Enhanced bioavailability C_max_ 3.1fold higher than free drug NMDA and AMPA receptors	[112]
BSA	Asiatic acid	GSH (EDAC coupling)	After NP preparation	Asiatic acid in ethanol (desolvant)	_	Enhanced bioavailability Cmax 1.6 fold higher than free drug T1/2 1.7-fold higher NMDA and AMPA receptors	[139]
BSA	Procyanidin C1	Glucose	Before NP preparation	C1 in ethanol (desolvant)	IV	GLUT1-mediated BBB transport;	[140]
HSA	Indirubin	Ethylenediamine	During desolvation/nab	During desolvation/nab	_	Improvement of DLC for hydrophobic drug	[141]
BSA	Fluorescent dye	Aromatic resuscitation drugs	Before NP preparation	Dye in DMSO added to BSA solution	IV	MeB−DIR appeared specifically in the pineal recess and cerebellar cortex.	[142]
BSA	Amphotericin B	BSA-binding peptide; MMP-3-responsive linker	Before NP preparation	AmB in oil phase pre-emulsion	IV	Adequate accumulation of drugs at target organs and cells Reduced lung/brain CFU (2 log_10_ & 1.5 log_10_)	[143]
HSA	methylprednisolone	NEP1-40 peptide	After NP preparation	After NP preparation	IV	Avoid side effect and Frequent injection sustained and significantly slower than the releas	[144]**.**
HSA	pGL3 plasmid	Linear PEI (22 kDa)	pGL3-PEI added post-NP	pGL3-PEI added post-NP	_	highly taken up into an *in vitro* Cb cell line via endocytosis	[109]
HSA	(DOX	Ethylenediamine; Mannopyranoside	Before NP preparation	DOX in desolvation solvent	IV	IC_50_ = 0.50 μg/mL (4-fold lower vs. plain NP); tumor growth 2-fold lower	[145]
HSA	GA	PEI	After NP preparation	GA added to HSA solution pre-NP synthesis	_	Enhanced stability for negative drug (negative like HSA) Increase zeta potential	[113]
BSA	PTX	Cholesterol	Before NP preparation	During self-assembly (in ethanol)	IV	improved stability and DLC	[146]
HSA	–	PEG	After NP preparation		IV	half-life was four times larger	[150]
BSA	DOX	PCB, PMPC, or PEG	Pre/post-NP functionalization	Post-functionalization	IV	PEG-grafted nanoparticles showed superior biodistribution PMPC-decorated polymer have better tumor accumulation	[151]
HSA	Curcumin	GSH, Erythrocyte membrane, DSPE-PEG3400-T807	Post-NP modification	Curcumin in ethanol (desolvant)	Tail vein injection	stable in the serum brain targeting (the only one reach to brain)	[152]
BSA	Tacrine hydrochloride	beta-Cyclodextrin derivatives	CD in BSA pre-desolvation	Post-NP soaking	intranasal	Avoid first pass effect to increase the half-life	[160]
HSA	Sulforhodamine B	Chitosan	After NP preparation	Drug added to HSA solution pre-NP	intranasal	Avoid first pass effect to strengthen the efficacy	[161]
HSA	Meloxicam	Poloxamer 407 (P407)	After NP preparation	Pre-NP	intranasal	Avoid first pass effect to improve drug absorption.	[162]

### 8.1 Technical and methodological limitations

Despite advances in functionalized AlNPs for brain delivery, key technical and methodological challenges limit robust outcomes. Variability in BBB penetration across and within studies arises from BBB heterogeneity, age, disease, genetics, and inconsistent target receptor expression, complicating universal targeting strategies. Ensuring batch-to-batch consistency in nanoparticle synthesis is difficult, as small changes in conditions affect size, drug loading, and ligand density, while the lack of standardized protocols hampers cross-laboratory comparisons. Quantitative reporting is often inconsistent, with relative rather than absolute measures of brain uptake, and diverse *in vitro* BBB models add to confusion about predictive value. Few studies provide direct head-to-head comparisons of functionalization approaches, limiting insight into the most effective strategies for clinical translation.

### 8.2 Safety concerns

While albumin is generally considered biocompatible and non-immunogenic, the functionalization of AlNPs can introduce new safety considerations. Long-term immunogenicity is a potential concern, particularly for nanoparticles functionalized with large proteins or peptides, which could elicit an immune response after repeated administration. Although many studies report no acute toxicity, the long-term effects of chronic exposure to these nanoparticles are not well understood. The Polysorbate 80 safety debate is another important consideration. While polysorbate 80 is widely used as a surfactant to enhance BBB penetration, there are concerns that it may cause transient disruption of the BBB, potentially allowing harmful substances to enter the brain. Although studies like those by Zensi et al. have shown that TJ integrity is maintained with some functionalized nanoparticles, the potential for off-target effects with polysorbate 80-coated nanoparticles warrants further investigation. Finally, the potential toxicity of cationic modifications must be carefully evaluated. Cationic polymers like PEI are often used to enhance cellular uptake and endosomal escape, but they can also induce cytotoxicity and membrane damage. While some studies have demonstrated that these modifications can be made safely, a thorough assessment of their long-term biocompatibility is necessary before clinical translation.

### 8.3 Clinical translation barriers

The clinical translation of functionalized AlNPs faces major obstacles. Scalability and GMP-compliant manufacturing are challenging, as many preclinical methods are difficult to scale and quality control is complex and costly. Regulatory approval is also demanding: agencies require stringent characterization and safety data for nanomedicines, and the long-term fate of nanoparticles remains a concern. High development costs and long, expensive clinical trials, especially for CNS diseases, raise questions about cost-effectiveness. To date, only limited clinical trial data exist for functionalized AlNPs in brain delivery. While Abraxane® sets a precedent for albumin-based nanomedicines, translating this success to CNS indications is substantially more challenging and remains to be demonstrated.

### 8.4 Future perspectives and recommendations

To realize the full clinical potential of functionalized AlNPs, future research must move beyond proof-of-concept studies and focus on addressing the limitations and challenges outlined above. A key priority is the standardization of fabrication and characterization protocols to enhance reproducibility and facilitate meaningful comparisons between studies. The development of more predictive and physiologically relevant *in vitro* and *in vivo* models of the human BBB is also crucial for improving the translation of preclinical findings. Future work should also focus on the development of multifunctional and theranostic nanoparticles that combine targeting, imaging, and therapeutic capabilities. The use of biodegradable linkers and stimuli-responsive release mechanisms can further enhance the safety and efficacy of these systems. Moreover, the exploration of personalized medicine approaches, such as selecting ligands based on the individual BBB phenotype of a patient, could lead to more effective and targeted therapies. Finally, a concerted effort between nanotechnologists, neuroscientists, clinicians, and regulatory agencies is essential to facilitate the translation of AlNP platforms from promising research tools to revolutionary treatments for some of the most challenging neurological disorders. By systematically addressing the technical, safety, and regulatory hurdles, we can unlock the full potential of functionalized AlNPs to transform the treatment of CNS diseases.

## CRediT authorship contribution statement

**Hanan Mohammad:** Writing – review & editing, Writing – original draft, Visualization. **Maher Darwish:** Writing – review & editing, Writing – original draft. **Gábor Katona:** Writing – review & editing, Writing – original draft, Supervision, Funding acquisition. **Ildikó Csóka:** Writing – review & editing, Writing – original draft, Supervision, Funding acquisition, Conceptualization.

## Declaration of competing interest

The authors declare that they have no known competing financial interests or personal relationships that could have appeared to influence the work reported in this paper.

## Data Availability

Data will be made available on request.
